# DNA polymerase IV primarily operates outside of DNA replication forks in *Escherichia coli*

**DOI:** 10.1371/journal.pgen.1007161

**Published:** 2018-01-19

**Authors:** Sarah S. Henrikus, Elizabeth A. Wood, John P. McDonald, Michael M. Cox, Roger Woodgate, Myron F. Goodman, Antoine M. van Oijen, Andrew Robinson

**Affiliations:** 1 School of Chemistry, University of Wollongong, Wollongong, Australia; 2 Department of Biochemistry, University of Wisconsin-Madison, Madison, Wisconsin, United States of America; 3 Laboratory of Genomic Integrity, National Institute of Child Health and Human Development, National Institutes of Health, Bethesda, Maryland, United States of America; 4 Departments of Biological Sciences and Chemistry, University of Southern California, Los Angeles, California, United States of America; University of Michigan, UNITED STATES

## Abstract

In *Escherichia coli*, damage to the chromosomal DNA induces the SOS response, setting in motion a series of different DNA repair and damage tolerance pathways. DNA polymerase IV (pol IV) is one of three specialised DNA polymerases called into action during the SOS response to help cells tolerate certain types of DNA damage. The canonical view in the field is that pol IV primarily acts at replisomes that have stalled on the damaged DNA template. However, the results of several studies indicate that pol IV also acts on other substrates, including single-stranded DNA gaps left behind replisomes that re-initiate replication downstream of a lesion, stalled transcription complexes and recombination intermediates. In this study, we use single-molecule time-lapse microscopy to directly visualize fluorescently labelled pol IV in live cells. We treat cells with the DNA-damaging antibiotic ciprofloxacin, Methylmethane sulfonate (MMS) or ultraviolet light and measure changes in pol IV concentrations and cellular locations through time. We observe that only 5–10% of foci induced by DNA damage form close to replisomes, suggesting that pol IV predominantly carries out non-replisomal functions. The minority of foci that do form close to replisomes exhibit a broad distribution of colocalisation distances, consistent with a significant proportion of pol IV molecules carrying out postreplicative TLS in gaps behind the replisome. Interestingly, the proportion of pol IV foci that form close to replisomes drops dramatically in the period 90–180 min after treatment, despite pol IV concentrations remaining relatively constant. In an SOS-constitutive mutant that expresses high levels of pol IV, few foci are observed in the absence of damage, indicating that within cells access of pol IV to DNA is dependent on the presence of damage, as opposed to concentration-driven competition for binding sites.

## Author summary

Translesion DNA polymerases play a critical role in DNA damage tolerance in all cells. In *Escherichia coli*, the translesion polymerases include DNA polymerases II, IV, and V. At stalled replication forks, DNA polymerase IV is thought to compete with, and perhaps displace the polymerizing subunits of DNA polymerase III to facilitate translesion replication. The results of the current fluorescence microscopy study challenge that view. The results indicate that DNA polymerase IV acts predominantly at sites away from the replisome. These sites may include recombination intermediates, stalled transcription complexes, and single-stranded gaps left in the wake of DNA polymerase III replisomes that re-initiate replication downstream of a lesion.

## Introduction

Translesion synthesis (TLS) DNA polymerases are produced at elevated levels in bacteria as part of the SOS response to DNA damage [1]. They have historically been thought to serve as a last resort DNA damage-tolerance mechanism, re-starting replication forks that have stalled at damage sites on the DNA [1–7]. TLS polymerases are highly error prone: inducing their activities leads to increased rates of mutation (error rates of up to 1 in every 100 nucleotides incorporated into DNA). TLS is an important source of mutations that fuel bacterial evolution [8–13]. For several species of bacteria, deleting genes for TLS polymerases dramatically reduces rates of antibiotic resistance development in laboratory measurements, and in some cases even reduces infectivity [9,14–22]. Many of the drugs used to treat bacterial infections cause an increase in mutation rates as a result of TLS [16]. It remains unclear, however, whether TLS polymerases contribute to resistance by providing damage tolerance, increasing cell survival and thus the chances that a resistant mutant will be found, or by facilitating adaptive mutation–selectively increasing mutation rates to speed the evolution of drug resistance [14–19].

DNA polymerase (pol) IV is thought to be the most abundant TLS polymerase in *E*. *coli*. From Western blots, it has been estimated that levels of pol IV increase from approximately 250 molecules per cell in the absence of DNA damage, to 2500 molecules per cell upon activation of the SOS damage response [23,24]. Pol IV promotes TLS on a variety of different lesion-containing DNA substrates, although its tendency for misincorporation varies with lesion type [25–32]. Pol IV bypasses adducts to the N^2^ position of guanines and a variety of alkylation lesions in a mostly error-free fashion [28–30,33–35]. When overexpressed, pol IV induces -1 frameshift mutations in cells treated with alkylating agents [36]. In addition to these lesion bypass activities, pol IV participates in transcription [37–40] and double strand break-repair repair [41–45], and contributes significantly to cell fitness in late stationary phase cultures in the absence of any exogenous DNA damage [8]. Pol IV is also reported to be required for formation of adaptive point mutations in the *lac* operon and was found to be a major determinant in the development of ciprofloxacin resistance in a laboratory culture model [9,46].

Visualisation of pol IV within live bacterial cells would make it possible to better understand how pol IV activity is regulated in response to DNA damage and test proposed models for its TLS activity at replisomes. Here, we report a single-molecule time-lapse approach to investigate pol IV dynamics and kinetics in live *E*. *coli* cells under normal growth conditions and following treatment with the antibiotic ciprofloxacin, the DNA-damaging agent MMS, or ultraviolet (UV) light. Our analysis indicates that most pol IV molecules carry out DNA synthesis predominantly outside replisomes and that access of pol IV to DNA is governed by more than simple concentration-action driven polymerase exchange.

## Results

### Construction and validation of a chromosomal *dinB-YPet* fusion

**Fig 1:**
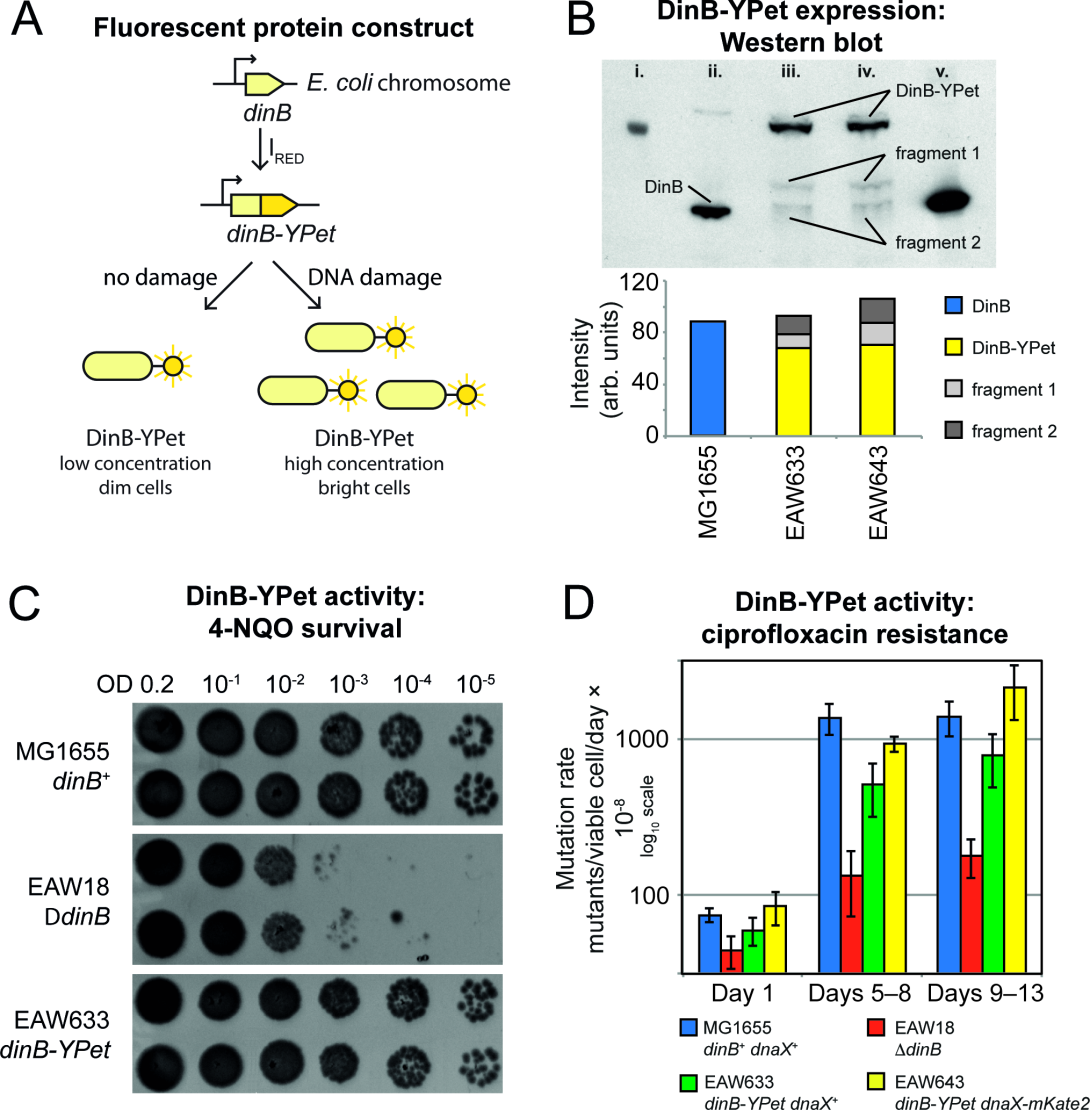
Construction of *E*. *coli* cells expressing labelled pol IV and analysis of bypass and mutagenic functions. (A) Construction of EAW633 (*lexA*^*+*^ *dinB-YPet*). The *dinB* gene of *E*. *coli* K12 MG1655 was modified using λ_RED_ recombineering so that pol IV is expressed as a fusion with the bright yellow fluorescent protein YPet (DinB-YPet). (B) Confirmation of DinB-YPet expression in ciprofloxacin-treated cells. (Upper part) Western blot of extracts from cells (treated with 30 ng/ml ciprofloxacin for 120 min), developed using anti-DinB antibodies. Lanes: i) molecular weight marker; ii) MG1655; iii) EAW633 (*dinB-YPet*); iv) EAW643 (*dinB-YPet dnaX-mKate2*); v) BL21 pLysS pET-DinB (uninduced cell extract). Bands corresponding to full length DinB-YPet are clearly visible in lanes ii and iii. A small amount of two DinB-containing fragments are also visible. Fragment 1 corresponds to DinB+linker. Fragment 2 corresponds to DinB +/- one or two residues. (Lower part) Results of densitometry measurements for lanes ii–iv. DinB-YPet is expressed at levels equivalent to wild-type DinB, however ~20% is proteolysed within the cells. (C) DinB-YPet retains lesion bypass activity. Strains were grown to exponential growth phase (OD_600_ = 0.2), serial diluted, and spotted onto LB agar plates containing 8 μM of 4-nitroquinolone-1-oxide (NQO). Because of an inability to bypass lesions induced by NQO, cells lacking *dinB* are sensitized by 3 orders of magnitude relative to wild type cells. Cells expressing DinB-YPet survival to levels equivalent to wild-type cells, indicating that DinB-YPet retains full lesion bypass activity. (D) DinB-YPet facilitates mutation to ciprofloxacin resistance. Approximately 10^8^ log-phase cells were spread onto LB agar plates containing 40 ng/ml ciprofloxacin and incubated at 37°C for 13 days. Colonies appearing on the plates were counted on days 4, 8 and 13. The number of new colonies appearing between each interval was determined and normalised against viable cell counts, as described in reference [9]. Cells lacking *dinB* produced only 10% as many ciprofloxacin-resistant colonies as wild-type cells. DinB-YPet expressing cells produced similar number of resistant colonies as wild-type cells, indicating that DinB-YPet supports mutagenic pol IV activities.

To visualise time-dependent changes in pol IV activity in response to DNA damage, we constructed an *E*. *coli* strain in which pol IV is fluorescently labelled, then imaged the resulting cells on a purpose-built single-molecule fluorescence microscope [47]. We created the pol IV-labelled strain in two steps. We started with a plasmid-based *dinB-eYFP* construct, shown previously to be active for pol IV-dependent DNA damage tolerance and mutagenesis by Mallik *et al*. [30]. We first replaced the gene for eYFP with the gene for the similar, but brighter, fluorescent protein, YPet. We then replaced the native *dinB* gene on the *E*. *coli* K12 MG1655 chromosome with the *dinB-YPet* fusion gene using λ_RED_ recombineering to create the strain EAW633. These cells express pol IV from its natural promoter, at its native chromosomal locus, but with YPet fused to its C-terminus through a twenty-amino acid linker (**Fig 1A**). To facilitate two-colour imaging of pol IV and replisomes, we also produced two strains with DNA polymerase III holoenzyme (pol III HE) markers. These strains expressed red fluorescent protein fusions of the pol III HE τ-subunit (EAW643; *dnaX-mKate2 dinB-YPet*) and ε-subunit (EAW641; *dnaQ-mKate2 dinB-YPet*) respectively. We have previously used *dnaX-YPet* and *dnaQ-YPet* fusions to indicate the position of replisomes [47]. Both the *dnaX-mKate2* and *dnaQ-mKate2* alleles used here are fully functional, having no impact on the growth of cells and showing no tendency for fluorescent protein-induced aggregation [48].

The expression and activity of the DinB-YPet fusion protein was verified using a series of three assays. First, we carried out Western blots using anti-DinB antibodies in order to compare the expression levels of DinB-YPet to those of untagged DinB (pol IV) in wild-type cells (**Fig 1B, S1 Fig**). In cells treated with ciprofloxacin, DinB-YPet is expressed at levels equivalent to wild-type DinB, although a small amount (~20%) is proteolysed to two shorter fragments within the cells. The larger fragment is probably produced via cleavage between the linker sequence and YPet, yielding YPet and DinB-linker. The smaller fragment migrates similarly to DinB and is probably produced via cleavage between the linker and DinB, yielding DinB and linker-YPet.

We next exposed cells to the DNA damaging agent 4-nitroquinoline-1-oxide (NQO) and measured survival using plate-based dilution assays (**Fig 1C**). As has been observed previously [29,44], cells lacking pol IV (Δ*dinB*) were much more sensitive to NQO than wild-type cells. Cells expressing the DinB-YPet fusion (EAW633) showed similar survival as wild-type cells, indicating that DinB-YPet retains pol IV-dependent lesion bypass activity.

When plated on LB agar containing an inhibitory concentration of the antibiotic ciprofloxacin, *E*. *coli* cells produce colonies of resistant mutants over the course of 13 days [9]. It was found previously that cells lacking pol IV activity give rise to fewer resistant mutants than wild-type cells [9]. We repeated these measurements and found that cells lacking pol IV (Δ*dinB*) produced only 10% as many ciprofloxacin-resistant mutants as wild-type cells (**Fig 1D**). Cells expressing DinB-YPet (EAW633 and EAW 643) produced similar numbers of resistant mutants as wild-type cells, indicating that DinB-YPet also remains active for pol IV-dependent mutagenesis.

### Direct observation of pol IV activity during the SOS response

**Fig 2:**
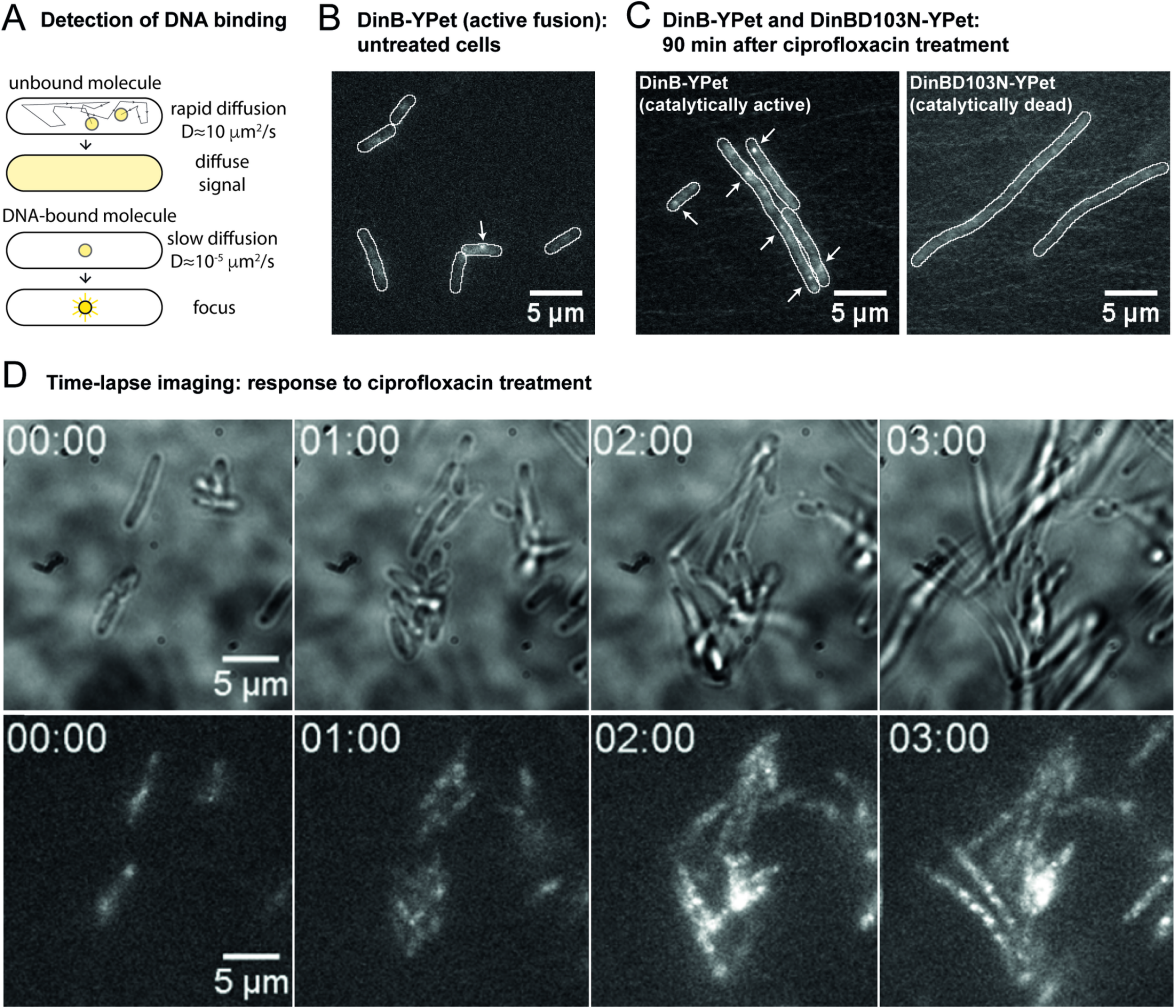
Single-molecule imaging of cells expressing DinB-YPet. (A) Detection of DNA-bound molecules in single-molecule images. Molecules of DinB-YPet that are not bound to DNA will diffuse quickly (D ≈ 10^5^ μm^2^/s for a typical cytosolic protein) and thus signals from individual molecules will blur over the entire cell in our images (exposure time = 30–100 ms). Molecules of DinB-YPet that are bound to DNA, however, experience greatly reduced motion and thus appear as punctate foci. Because of this diffusional contrast, it is possible to detect individual molecules of DinB-YPet when bound to DNA. (B) Single-molecule sensitive fluorescence image of undamaged EAW643 cells showing low-level DinB-YPet signals at 50 ms exposure time. (C) Average projection of rapid acquisition (effective exposure time 306 ms) for DinB-YPet (left) and DinBD103N-YPet (right). (D)Time-lapse imaging of pol IV up-regulation in response to ciprofloxacin treatment. Images shown are a montage of a three-hour time-lapse series. Cells were initially grown in rich medium in the absence of exogenous DNA damage. At t = 0 min, the flow cell inlet was switched to medium containing 30 ng/ml ciprofloxacin. At each field-of-view, a bright-field image and a DinB-YPet fluorescence image were collected every 5 min for 180 min. Time stamps indicate hours after ciprofloxacin addition.

We imaged EAW643 cells in the context of home-built flow-cells, which enable continuous flow of media throughout our measurements. For this study, we recorded two types of fluorescence movies: rapid-acquisitions, which capture the motions of molecules on the milliseconds–seconds timescale; and time-lapse measurements, which capture changes in pol IV behaviour over the course of hours. Single-molecule level measurements allow us to observe binding of pol IV molecules to DNA. On our imaging timescale (34 ms exposures), proteins moving freely through the cytosol diffuse quickly (*D* ≈ 10^2^ μm^2^/s) and thus appear as a blur (**Fig 2A)**. Any pol IV molecules bound to specific binding sites on the DNA, however, should move much more slowly; their motion will be dictated by the motion of the binding site. In *E*. *coli*, individual sites on the chromosome have an apparent diffusion constant *D* ≈ 10^−5^ μm^2^/s [49]. As pol IV requires ~100 ms to incorporate a single nucleotide, we expect that any molecules synthesising DNA will appear relatively static in our images and thus produce bright foci.

In the absence of damage, we observed small, but measureable DinB-YPet signals within cells, consistent with continuous low-level production of pol IV (**Fig 2B**). It is possible to calibrate the fluorescence intensities of cells against the intensity of individual molecules in order to determine the number of molecules in each cell (**see Experimental Procedures**). We calculated that EAW643 cells express 20 ± 3 molecules of DinB-YPet per cell (STD = 36; *n* = 105 cells) in the absence of damage. Using cell size parameters measured from bright-field images it is further possible to determine the volume of each cell, and subsequently to determine the DinB-YPet concentration. We calculate that in the absence of damage the DinB-YPet concentration is 6 ± 1 nM.

The pol IV levels measured here by microscopy are somewhat lower than previous estimates of 250 molecules of pol IV per cell, based on Western blots [24]. It has been demonstrated previously that under conditions similar to those used here that >90% of YPet molecules are in the mature, fluorescently active form [50]. The small amount of proteolysis of DinB-YPet observed in the Western blot (**Fig 1B**) would be expected to yield an intact YPet fragment. Thus, the microscopy-based measurements should still produce an accurate measure of DinB levels. At worst, DinB levels would be underestimated by ~20%. To probe this discrepancy further, we repeated the Western blot analysis (**S1 Fig**). The values we calculated varied considerably between replicates, reflecting the difficulties associated with quantifying Western blots of low abundance proteins. All values were, however, significantly lower than those determined in the Kim *et al*. study and were consistent with the fluorescence microscopy results. Taking the mean of two independent blots, the current Westerns indicate that MG1655 contain 33 molecules of DinB per cell on average. The strain used in the Kim *et al*. study, YG2247 (a derivative of P90C) returns similar value: 30 molecules per cell on average. DinB-YPet was measured at 19 molecules per cell. The fluorescence microscopy measurements presented here are much more sensitive, and far less variable, than Western blotting and likely to provide more accurate results. We therefore conclude that the value measured by fluorescence microscopy, 20 molecules of DinB per cell, is correct and that the original value of 250 was an overestimation [24].

In rapid acquisition movies, we observe that the DinB-YPet signal is primarily diffuse (**Fig 2B**): cells contain 0.5 ± 0.5 foci per cell on average (i.e. one focus for every two cells; STD 1.11; *n* = 105 cells). This measurement indicates that pol IV rarely binds to DNA in the absence of damage.

We then induced DNA damage by switching to medium containing 30 ng/mL ciprofloxacin, an antibiotic that inhibits DNA gyrase and forms covalent adducts on the DNA [51]. These inhibit DNA replication and lead to induction of the SOS response. Under these conditions, we observed that cells were longer and exhibited stronger DinB-YPet signals (**Fig 2C**). This observation is consistent with increasing production of pol IV as part of the SOS response, leading to higher concentrations of pol IV in the cell. Punctate foci were visible after ciprofloxacin addition, consistent with pol IV binding to DNA. Cells expressing a catalytically dead variant of pol IV [52,53], DinB(D103N)-YPet, did not produce foci when imaged under the same conditions (**Fig 2C**). We therefore conclude that ciprofloxacin treatment leads to a significant increase in the number of pol IV binding events on the DNA.

**Fig 3:**
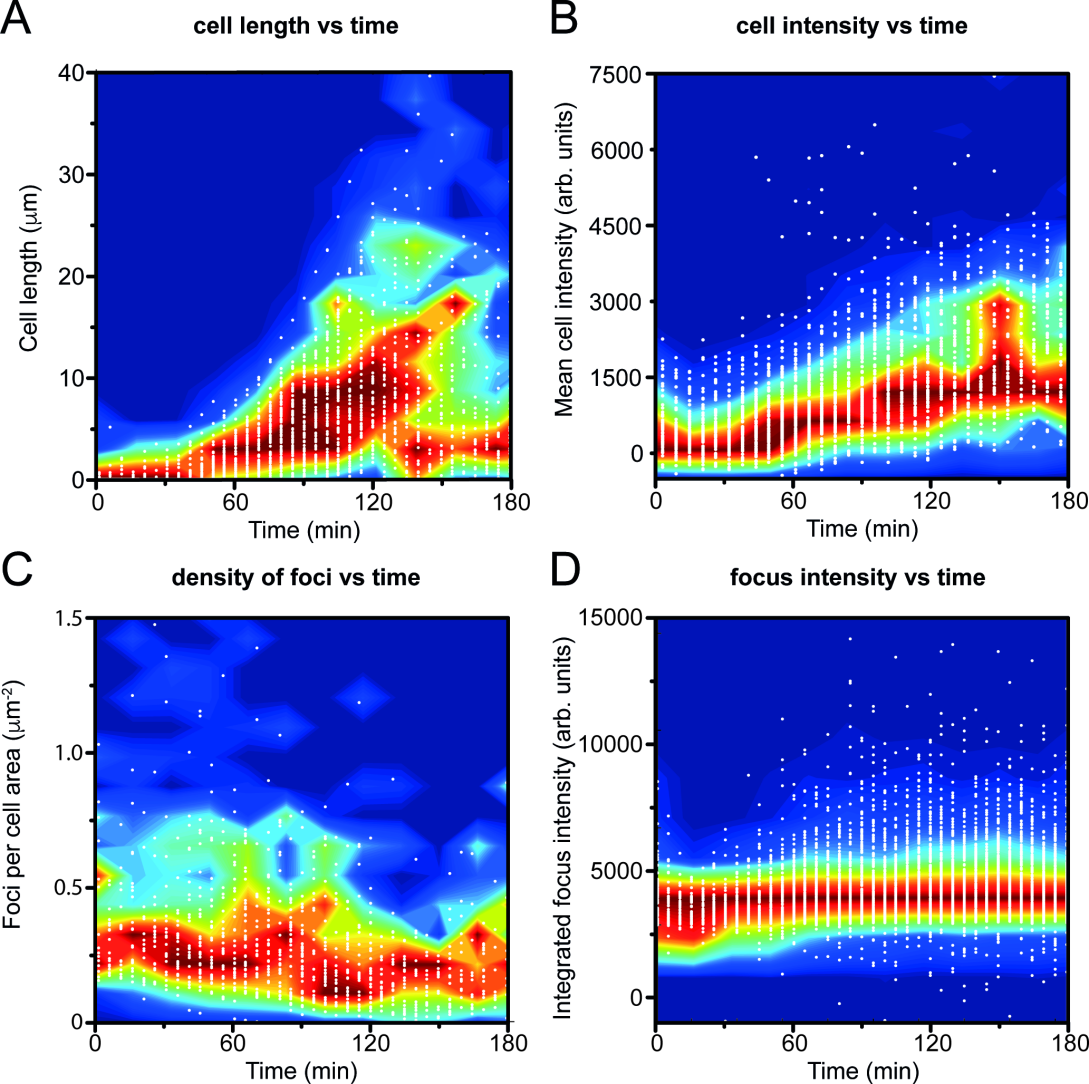
Scatter plots of cell-size and fluorescence signal parameters from time-lapse imaging of DinB-YPet cells treated with ciprofloxacin. White points indicate individual data-points, while blue-to-red contours indicate frequencies of observations. Blue areas indicate regions of the plot containing few data points; red areas indicate regions containing a large number of data points. Frequencies were normalised at each time-point to the maximum value at that time-point. (A) Distribution of cell lengths based on bright-field images, showing ciprofloxacin-induced filamentation. (B) DinB-YPet fluorescence per cell, measured as the mean pixel intensity within each cell, showing up-regulation of DinB-YPet. (C) Density of DinB-YPet foci, measured as the number of foci per cell area (μm^2^), showing the density remains relatively constant over the three-hour measurement. (D) Integrated fluorescence intensity of foci. Each focus was fit with a 2D Gaussian function; the volume under this function represents the integrated fluorescence intensity. Foci become brighter over the course of the measurement, indicating that a higher number of DinB-YPet molecules bind at each binding site. We conservatively estimate that >100 cells were used in each measurement.

Time-lapse analysis indicated that cells filament and exhibit a strong increase in DinB-YPet fluorescence, beginning approximately 20 min after the addition of ciprofloxacin (**Fig 2D**; **Fig 3A and 3B, S1 Movie**). From 90–180 min, the DinB-YPet concentrations plateaus. We calculate that at this point, cells contain an average of 279 ± 33 DinB-YPet molecules per cell (STD 28; *n* = 105 cells). Thus, ciprofloxacin-treated cells contain 14 times more molecules of DinB-YPet than undamaged cells. Due to damage-induced filamentation, however, the ciprofloxacin-treated cells are 2.5 times larger in volume. Thus, the concentration of DinB-YPet after treatment with ciprofloxacin is 34± 3 nM, is only 5.5 times higher than in the absence of damage. The number of pol IV molecules per cell that we measure by microscopy after ciprofloxacin treatment is lower than previous estimates of pol IV expression (~2500 molecules per cell) based on Western blotting of MMS-treated cells [24]. Values measured by Western blot during the current study were highly variable, but all were significantly lower than the previous estimate of 2500 molecules per cell. The values measured here, ~100 molecules per cell following treatment with 30 ng/ml ciprofloxacin for 2 h, are more consistent with those measured by fluorescence microscopy (**S1 Fig**). We conclude that the value originally published by Kim *et al*. is likely to be overestimated. Based on the microscopy results, which are likely to be more accurate than those of Western blots, we concluded that there are 250 molecules of DinB per cell following ciprofloxacin treatment.

We next measured as a function of time the number of DinB-YPet foci per cell area (i.e. the density of foci throughout the cell) and their intensities. The density of DinB-YPet foci in cells remained relatively constant (**Fig 3C**), whereas the intensities of foci increased over time (**Fig 3D**), following a similar trend as the increase in pol IV concentration (**Fig 3B)**. These observations indicate that the number of binding sites for pol IV in each cell remains relatively constant from 30–180 min after ciprofloxacin addition, whereas the number of molecules bound at each binding site increases in time. Comparing the intensities of DinB-YPet foci to the intensity of a single YPet molecule, we calculate that in the early stages of the response (30–90 min, foci contain 1–2 DinB-YPet molecules while in the later stages (90–180 min), foci contain 2–4 molecules (**S2 Fig**).

### Colocalisation between pol IV and replisomes

Two models have been proposed for pol IV activity in the vicinity of replisomes. In the first and most widely cited model, pol IV acts within the replisome [1–7]. Here the pol IV exchanges with a pol III that has stalled at a lesion in the template, bypasses the lesion, then exchanges back out of the replisome, allowing pol III to continue with processive DNA synthesis. In the second model pol IV carries out postreplicative TLS at gaps left in the wake of replisomes that skip over lesions [54–56]. In principal both mechanisms could be at play within cells. In addition to these (near) replisomal activities, a number of studies have implicated pol IV in a variety of other cellular processes, including transcription and recombination [30,37–45].

To further examine the activities of pol IV inside and outside the replisomal context, we imaged both DinB-YPet and a replisome marker, either DnaX*-*mKate2 or DnaQ-mKate2, which allowed us to visualise the position of pol III HE complexes. The DnaX (τ-subunit) and DnaQ (ε-subunit) proteins are stably associated within the pol III HE in *E*. *coli*; they do not exchange in and out of the complex [48]. We assume that foci formed by DnaX-mKate2 and DnaQ-mKate2 exclusively indicate the positions of pol III HE complexes acting within replisomes. From this point, we make reference to replisome markers and replisome foci. These refer to DnaX-mKate2 foci unless otherwise stated. As the pol III HE contains (at least) two polymerases, we expect that if pol IV exchanges with one of the pol III cores, pol III HE will remain bound and the pol IV and replisome markers will colocalise. On the other hand, if pol III HE does fully dissociate from the DNA as pol IV binds at the replication fork, the DinB-YPet and replisome foci would not colocalise. In this case we would expect that as the number of DinB-YPet foci in cells increased, there would be a significant decline in the number of replisome foci.

**Fig 4:**
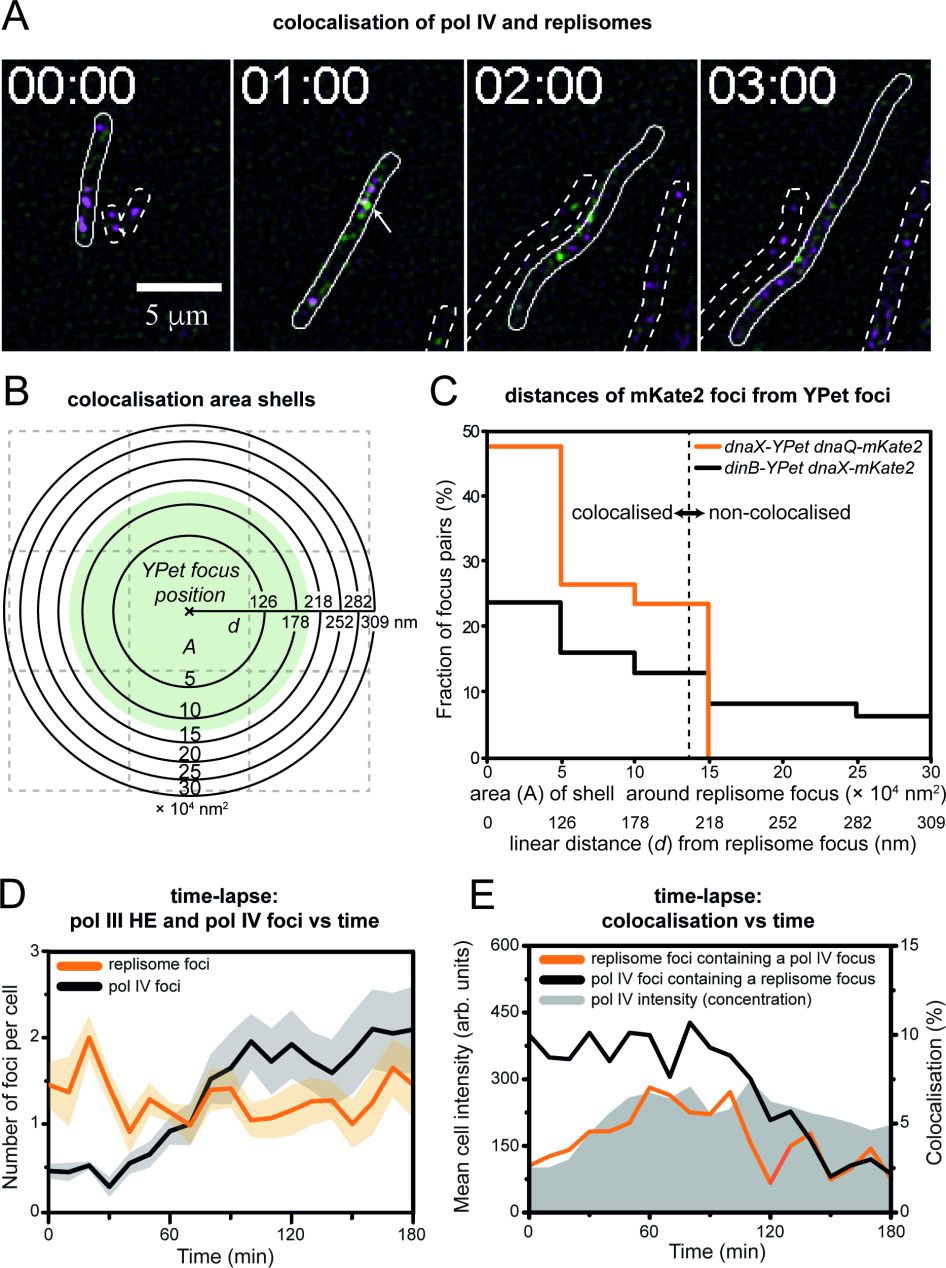
Colocalisation of pol IV with replisomes. (A) Montage of two-colour time-lapse movie recorded after treatment 30 ng/mL ciprofloxacin. Pol IV (DinB-YPet) foci appear green and replisome (DnaX-mKate2) foci appear in magenta. Colocalised foci appear white. For display purposes, images were subjected to spatial filtering to enhance foci [47]. (B-C) Analysis of colocalisation distances for foci detected in two-colour images. (B) Diagram of area shells used for colocalisation analysis. As colocalisation is a radial measurement, histograms of colocalisation distances are constructed using bins of linearly increasing area rather than distance. (C) Histograms of colocalisation distances for foci within a doubly labelled replisome strain (EAW203; *dnaX-YPet dnaQ-mKate2*) and a two-colour pol IV/replisome strain (EAW643; *dinB-YPet dnaX-mKate2*). As expected, distances between DnaX-YPet and DnaQ-mKate2 foci fall within a narrow distribution, indicative of ‘tight’ colocalisation. Distances between DinB-YPet and DnaX-mKate2 foci present a much broader distribution, indicative of ‘loose’ colocalisation. (D) Plot of the number of pol IV and replisome foci per EAW643 cell as a function of time. Data were compiled from ten technical replicates. Shaded areas indicate the standard error of the mean between these replicates. Some cells were lost from the coverslip surface during the measurement. A total of 188 cells remained bound and were analysed over the full course of the measurement. (E) Plots of mean cell intensity and colocalisation between pol IV and replisome foci. The mean cell intensity (grey shaded area) is a direct measure of the pol IV concentration in cells. Foci located within 200 nm of each other were defined as being colocalised. Colocalisation was measured in two ways: the proportion of pol IV foci that contain a colocalised replisome focus (black line), and the proportion of replisome foci that contain a colocalised pol IV focus (orange line). Data were compiled from ten technical replicates. Shaded areas indicate the standard error of the mean between these replicates. The total number of cells analysed were not determined in these measurements. We conservatively estimate that >1000 cells were used in each measurement. The analysis includes a total of 17005 DnaX-mKate2 foci and 12408 DinB-YPet foci.

We recorded two-colour time-lapse movies and measured the number of replisome and pol IV foci as a function of time, as well as their colocalisation (**Fig 4**). Two forms of analysis were carried out. To further investigate whether pol IV acts within or behind replisomes, we measured pair-wise distances between pol IV foci and replisome markers. To investigate the balance between (near) replisomal and non-replisomal activities of pol IV, we measured time-dependent changes in the proportion of pol IV foci that tightly colocalised with replisome markers.

If pol IV carries out replicative TLS (within the replisome), one would expect to observe ‘tight’ colocalisation of pol IV foci with replisome foci; a histogram of pair-wise distances between DinB-YPet and DnaX-mKate2 foci would be expected to produce a relatively sharp peak. One might also expect enrichment of pol IV foci close to replisomes if pol IV instead carries out postreplicative TLS in gaps left behind the replisome. In this case however, replisomes would be expected to rapidly move away from gaps after they are created. This would lead to a type of ‘loose’ colocalisation that would manifest as a broad distribution of distances between pol IV foci and replisome markers.

We first measured pair-wise distances between foci in the strain EAW203 (*dnaX-YPet dnaQ-mKate2*) as a control. In this strain, the replisomes are labelled in two colours, producing a very high degree of colocalisation in two-colour images [48]. Pair-wise distances between DnaX-YPet and DnaQ-mKate2 foci were plotted as a histogram. As colocalisation is a radial measurement there is a higher probability of detecting pairs separated by longer distances because longer search radii will cover a larger area of the image. To account for this, we assigned histogram bins based on shells of regularly increasing area rather than binning by linear distances (**Fig 4B**). For the two-colour replisome strain, the histogram contained a sharp peak (**Fig 4C**). All mKate2 foci fell within 218 nm of a YPet focus (i.e. they fell within a 15 × 10^4^ nm^2^ area shell). The width of the peak reports on the colocalisation error, which is a product of the localisation errors associated with fitting the YPet and mKate2 foci and any sample motion that occurs in the interval between collecting images in each colour channel (~2 s). We then repeated the analysis for the two-colour pol IV/replisome strain. A histogram of pair-wise distances for pol IV and replisome foci showed a considerably broader peak (**Fig 4C**), indicative of ‘loose’ colocalisation. Together these observations suggest that many DinB-YPet foci form close to, but not at replisomes. Thus, the results of this analysis are consistent with pol IV carrying out postreplicative TLS. With the current data, it is not possible to determine if pol IV carries out postreplicative TLS exclusively, or if both replicative and postreplicative TLS occur.

We next analysed time-dependent changes in colocalisation behaviour. Based on the histogram of pair-wise distances for the two-colour replisome strain (**Fig 4C**), we defined foci detected in time-lapse analyses as being colocalised if their fitted centroid positions fell within 200 nm of each other. We found that following ciprofloxacin treatment the number of replisome spots in cells remained relatively constant over time, indicating that pol III HE was not being removed from replisomes to a large extent (**Fig 4D**). We determined colocalisation in both directions, i.e. we measured the proportion of DinB-YPet foci that overlapped with a replisome focus, as well as the proportion of replisome foci that overlapped with a pol IV focus. From 0–100 min after ciprofloxacin addition, 10% of pol IV foci colocalise with replisomes (**Fig 4E**), significantly above levels expected by chance (~5%, **see Experimental Procedures**), but well below levels expected if pol IV predominantly operates in the vicinity of replisomes. This observation suggests that the majority of pol IV’s activities could be non-replisomal (**see** Discussion). Additionally, we found that in the late stages of the SOS response there was an even higher proportion of non-replisomal pol IV foci: from 100 min the proportion of pol IV foci that colocalise with replisomes falls to just 2.5%. Similar behaviour is observed when measuring colocalisation in the other direction. From 0–60 min, the proportion of replisomes that contain pol IV increases to 7%, tracking the increase in pol IV concentration within that period. From 60–100 min, the colocalisation plateaus at this level (modestly above the level expected by chance), in line with a plateau in the pol IV concentration. From 100–180 min, however, the proportion of replisomes that contain pol IV falls sharply; the average colocalisation is ~3% between 110–180 min, close to levels expected by chance. In contrast, the pol IV concentration remains elevated during this period. For both replisomes and pol IV, a plot of the number of foci per cell shows no evidence of a sharp transition at 100 min (**Fig 4D**), ruling out the possibility that the drop in colocalisation (**Fig 4E**) results from sudden loss of replisome or pol IV foci. Throughout the first 100 min, the concentration of pol IV increases, whereas the proportion of pol IV foci that colocalise with a replisome marker remains relatively constant. This indicates that the proportion of pol IV molecules that bind near replisomes is independent of the pol IV concentration. Similar results were obtained using EAW641, in which replisomes are marked by expression of DnaQ-mKate2 rather than DnaX-mKate2 (**S3 Fig**).

To determine which DinB-YPet foci are likely to represent catalytically active molecules, we measured colocalisation using a longer (300 ms) exposure time. Pol IV molecules engaged in DNA synthesis will remain associated with the DNA for longer than molecules that bind non-productively to DNA or to other factors. In 300 ms images, foci are visible in DinB-YPet cells, but not in cells expressing catalytically dead DinB(D103N)-YPet (**Fig 2C**). In time-lapse images we detected fewer foci than when using 50 ms exposures (**S4 Fig**), however the proportion of foci that colocalised with replisomes in 300 ms images (5%; **S4 Fig**) was similar to that observed in 50 ms exposures (10%; **Fig 4E**). Furthermore, a similar drop in colocalisation at 100 min was observed. From 0–90 min, 5% of pol IV foci overlap with a replisome (**S4 Fig**). From 100–180 min, the colocalisation drops to 1.5%. Colocalisation of replisomes with pol IV shows a similar trend. From 0–90 min, 0.5% of replisomes have a pol IV focus, however, after 90 min only 0.2% of replisomes contain a pol IV focus. The fact that colocalisation was similar for both the 50 ms and 300 ms exposures indicates that there is no major difference in the lifetimes of foci formed near to, or away from replisomes and suggests that pol IV engages in DNA synthesis at sites both near to, and away from replisomes.

### Pol IV activity is not governed by mass action-driven competition

In light of the observation that the colocalisation of pol IV and replisomes does not track with pol IV concentration, it is unlikely that access of pol IV to different DNA substrates is governed by mass action-driven competition alone. To explore this issue further, we altered the expression levels of pol IV in two different ways and examined the effects on pol IV focus formation and colocalisation with replisomes. We first increased the amount of pol IV in cells by transforming SSH001 cells (Δ*dinB dnaQ-mKate2*) with the DinB-eYFP plasmid used by Mallik *et al* [30]. Within this plasmid, pol IV is expressed from its natural promoter. However, because the plasmid is maintained at ~5–10 copies per cell, pol IV levels are expected to be much higher than when it is expressed from the chromosome.

**Fig 5:**
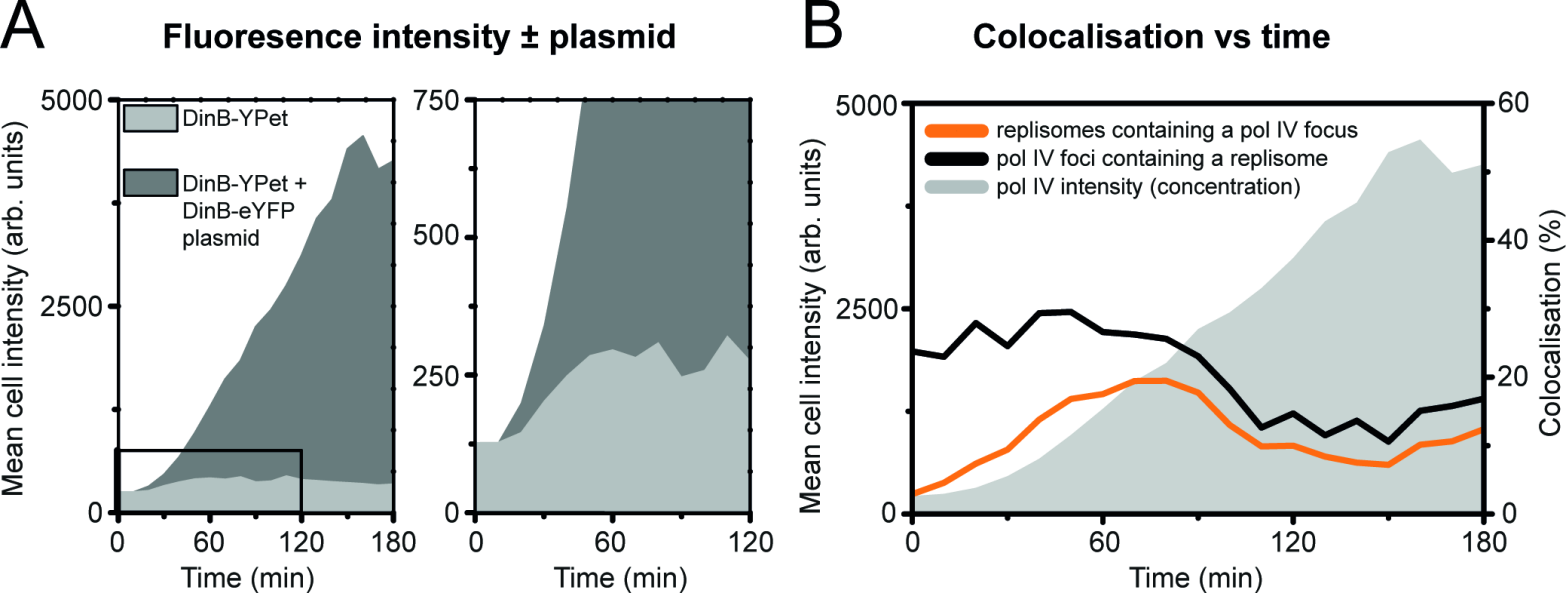
Colocalisation of pol IV with replisomes in the presence of additional fluorescently labelled pol IV expressed from a plasmid. (A) Mean cell intensity measurements for EAW643 cells (Pol IV^+^; light grey line) and EAW643 cells containing pPFB1188 (expressing additional DinB-eYFP from the *dinB* promoter; Pol IV^++^; dark grey line). Cells containing pFB1188 clearly express much higher levels of labelled pol IV, however because cells contain an unknown ratio two different YFPs (DinB-YPet and DinB-eYFP), it is not possible to measure the pol IV concentration. (B) Plots of mean cell intensity and colocalisation between pol IV (DinB-YPet/DinB-eYFP) foci and replisome (DnaX-mKate2) foci. The mean cell intensity (grey shaded area) is a convoluted measure of the combined DinB-YPet and DinB-eYFP concentrations in cells. Colocalisation was measured in two ways: the proportion of pol IV foci that contain a colocalised replisome focus (black line), and the proportion of replisome foci that contain a colocalised pol IV focus (orange line). Data were compiled from ten technical replicates. The total number of cells analysed were not determined in these measurements. We conservatively estimate that >500 cells were used in each measurement. The analysis includes a total of 27651 DnaX-mKate2 foci and 31978 DinB-YPet/DinB-eYFP foci.

We repeated the time-lapse analysis for this plasmid-containing strain and observed much higher levels of fluorescence (**Fig 5A**). We calculated that cells contain approximately 7000 molecules of DinB-eYFP after 90 min; already 14-fold higher than in cells expressing only DinB-YPet from the chromosome (280 molecules per cell) after >120 min. Despite this large change in the amount of pol IV, we observed the same time-dependent loss of colocalisation as before, although colocalisation in the initial stages of SOS was somewhat higher (**Fig 5B**). In the plasmid-containing strain, we found that the proportion of replisomes that contained a pol IV focus increased from 3 to 20% within the first 90 min after ciprofloxacin addition. Similarly, the proportion of pol IV that colocalise with a replisome focus is 25–30% from 0–90 min. We found that pol IV foci were noticeably brighter in the presence of the pol IV-expressing plasmid than in its absence, especially after 100 min (**S5A Fig**). We calculated that each focus contains ~3–10 molecules of pol IV, while at the later stages foci contain > 30 molecules of pol IV (**S5B Fig**). Cells that carried the *dinB-eYFP* plasmid only (i.e. they lacked a chromosomal copy of *dinB*) produced foci that showed similar levels of colocalisation with replisomes as cells that contained both *dinB-YPet* and *dinB-eYFP* (**S6 Fig**).

Importantly, we found that the *dinB-eYFP* plasmid is toxic to cells during the late SOS response. We observed that 17% of cells carrying the *dinB-eYFP* plasmid lysed upon ciprofloxacin treatment (**S7 Fig**). In comparison, <3% of wild-type MG1655 or EAW643 cells lysed during the measurements. We also noted that cells containing the DinB-eYFP plasmid elongated at a much slower rate than the either EAW643 lacking the plasmid or wild-type cells. These observations suggest that in the presence of the *dinB-eYFP* plasmid, pol IV reaches concentrations high enough above wild-type levels that it begins to interfere with cell growth.

**Fig 6:**
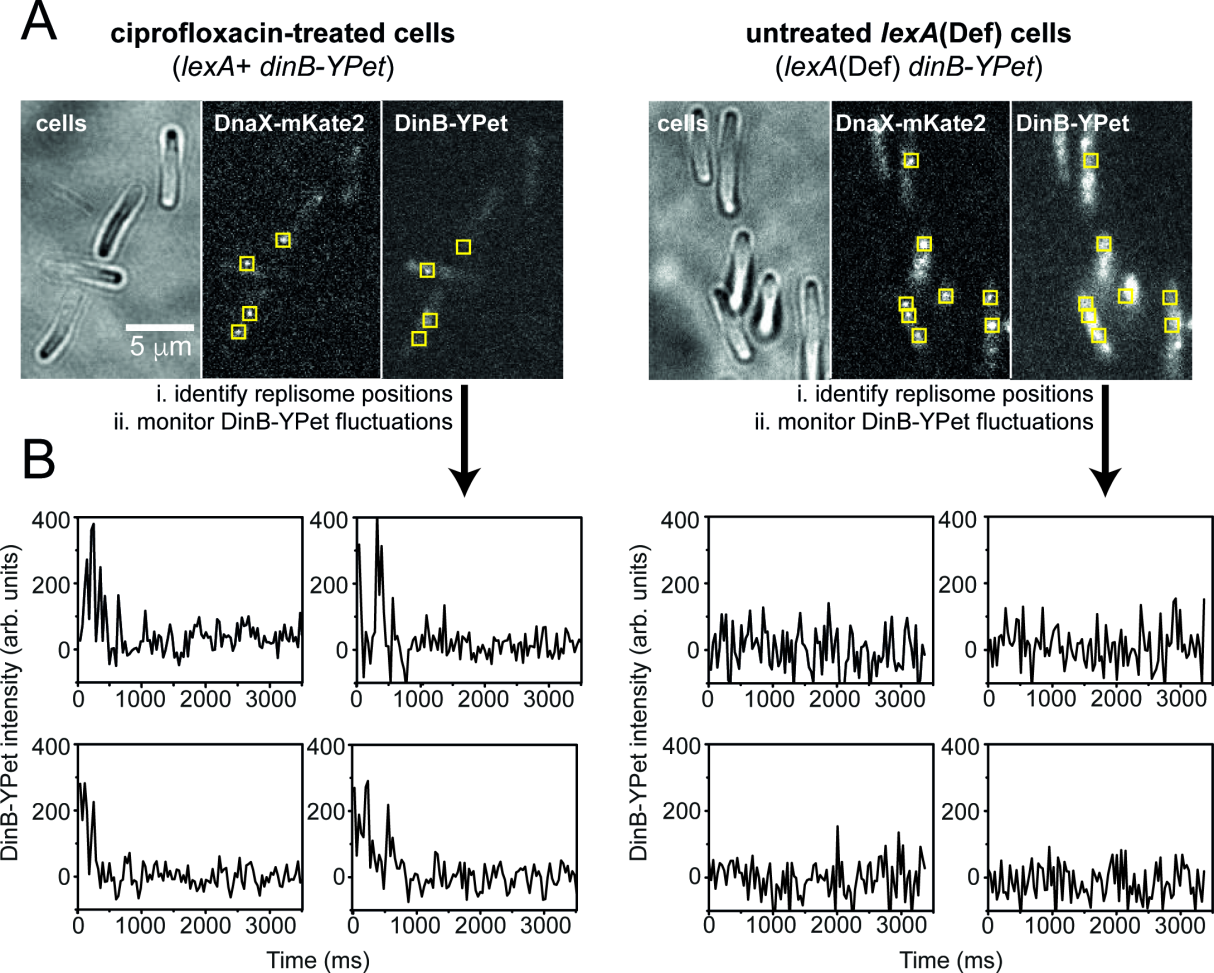
Comparison of DinB-YPet behaviour in untreated, *lexA*(Def) cells and ciprofloxacin-treated *lexA*^+^ cells. (A) Representative images of ciprofloxacin-treated *lexA*^+^ cells (left) and untreated *lexA*(Def) cells (right). (B) Representative intensity vs time trajectories for DinB-YPet signals in the vicinity of replisomes. Additional, randomly selected trajectories appear in **S7 Fig** (ciprofloxacin-treated *lexA*^+^ cells) and **S8 Fig** (untreated *lexA*(Def) cells). 5×5 pixel regions of interest were placed at replisome foci, then used to monitor fluctuations in DinB-YPet signals (see panel A). In ciprofloxacin-treated *lexA*^+^ cells, DinB-YPet signals are elevated in the vicinity of replisomes for multiple frames, indicating long-lived binding events. In untreated *lexA*(Def) cells no events are visible in which the DinB-YPet is elevated in the vicinity of replisomes for more than a single 34 ms frame, indicating no long-lived binding events.

We next examined pol IV behaviour in *lexA*(Def) cells. This background contains a mutation that inactivates the LexA repressor protein, causing cells to constitutively express high levels of all proteins within the SOS regulon, including pol IV [23,57,58]. To prevent cell death from constitutive SOS-driven filamentation, we also introduced a *sulA*^-^ mutation. The *lexA*(Def) background allowed us to investigate if high concentrations of pol IV allow it to bind to DNA in the absence of DNA damage. In the *lexA*(Def) background, we calculate the concentration of pol IV to be 96.5 ± 7.29 nM (STD 53.54 nM, *n* = 54 cells), 15.6 times higher than undamaged wild-type cells, and 2.8 times higher than wild-type cells treated with ciprofloxacin for 2h. The elevated concentrations of DinB-YPet in the *lexA*(Def) background created a high background of diffuse fluorescence signal, making it difficult to observe pol IV foci directly (**Fig 6A**). Instead, we recorded fluorescence movies at high time resolution as DinB-YPet photobleached. Once ~50% of the DinB-YPet had bleached, it was possible to observe foci. These foci, however, were extremely transient, rarely persisting beyond a single 34 ms frame, indicative of only short-lived events on the DNA. It appeared that very few of these transient foci colocalised with replisomes. To examine this more closely, we analysed time-dependent fluctuations in DinB-YPet signals at replisomes, and away from replisomes, and compared signals from undamaged *lexA*(Def) cells against signals from wild-type cells treated with ciprofloxacin (**Fig 6B; S8 and 9 Figs**). The trajectories indicate some transient binding of DinB-YPet at replisomes in the *lexA*(Def) strain, however these trajectories appear comparable to those for regions-of-interest placed outside of replisomes. In comparison, replisome trajectories in the ciprofloxacin-treated wild-type cells often indicated pol IV binding events lasting >1s before dissociation or photobleaching occurs. These observations clearly indicate that even the highest concentrations of pol IV that could naturally occur in cells at the height of the SOS response are not enough to allow pol IV to enter replisomes and productively synthesise DNA. Pol IV either requires DNA damage, or additional factors that accumulate in response to damage, to be recruited to DNA.

## Discussion

The work described here has four conclusions and two implications. We conclude that: i) most pol IV foci form outside of replisome regions; ii) pol IV foci often form near to, rather than at, replisomes; iii) pol IV is prevented from binding at regions of the DNA close to replisomes during the late stages of SOS (>90 min after SOS induction); and iv) replisome access is not improved substantially when pol IV is highly expressed. We infer from these observations that pol IV is mostly active outside of replisomes, promoting TLS in gaps formed in the wake of the replication fork and functioning in other, non-replisomal capacities. We further infer that DNA polymerase IV does not achieve access to the replisome via a simple mass action displacement of DNA polymerase III components. We elaborate on these themes below.

### Non-replisomal activities of pol IV

We observed that only 5–10% of pol IV foci tightly colocalise with replisome markers. Assuming that these foci indicate sites of pol IV binding (short- and long-lived binding events) to the DNA, this observation implies that the vast majority of pol IV molecules could work on other, as yet unidentified substrates. What other DNA structures might pol IV work at? Do the mutagenic and non-mutagenic lesion-bypass activities of pol IV relate to its action at replisomes, as is often assumed, or do they relate to activities at other DNA structures? Pol IV has been previously found to be involved to a range of different pathways, including rescue of stalled transcription complexes [38], double-strand break repair [30,59], adaptive mutation [46,60] and stationary phase fitness [8]. It is possible that the non-replisomal DinB-YPet foci that we observe represent pol IVs participation in these pathways. Determining how pol IV activity is distributed amongst these various pathways is far beyond the scope of this study. It is clear, however, that two-colour fluorescence imaging has a large part to play in characterising the range of substrates used by pol IV in cells.

### Replisome-proximal activities of pol IV: TLS is predominantly postreplicative

The minority of pol IV foci that do form near replisomes show only loose colocalisation: there is a very broad distribution of distances between pol IV foci and replisomes. This result is inconsistent with the notion that pol IV-dependent TLS exclusively takes place at replications forks that have stalled at a damage site on the template DNA [1–7]. The results strongly suggest that pol IV is capable of carrying out post-replicative TLS within gaps behind the fork. The results do not indicate, however, whether pol IV acts purely in a post-replicative sense, or whether both replicative and post-replicative TLS are possible. Although the DinB-YPet fusion behaves like wild-type pol IV in the NQO-survival and ciprofloxacin resistance assays, we cannot formally rule out the possibility that the addition of YPet to pol IV somehow alters the balance between TLS at replication forks vs TLS within gaps.

There is a well-established, and growing, body of literature that points to replisomal lesion skipping as a major mechanism of DNA damage tolerance in bacteria [54–56; 61–68]. The idea that pol IV participates in post-replicative TLS is consistent with the lesion-skipping scenario, as proposed previously [69–72]. Rather than replisomes stalling when they encounter lesions, they simply re-prime the template and continue synthesis downstream of the lesion. In its wake, the replisome leaves a lesion-containing single stranded DNA gap. Such gaps could not be repaired by pathways that work on double stranded DNA, such as nucleotide excision repair, and would instead be initially bypassed, either by TLS, or by *recFOR*-mediated daughter strand gap repair. Based on a lack of colocalisation with replisome markers, we have previously hypothesised that another TLS polymerase, pol V, also carries out post-replicative TLS in single stranded DNA gaps [47]. It would of considerable interest to determine if the third TLS polymerase in *E*. *coli*, pol II, also shows loose colocalisation with replisomes in cells carrying DNA damage.

### Pol IV does not access replisomes through mass action-driven exchange with pol III HE

A conventional view has been that pol IV gains access to replisomes upon SOS induction because it is produced at higher concentrations, allowing it to better compete with pol III HE for binding to replication forks [1–7,54]. Observations made during the current study are inconsistent with this simple mass action-driven mechanism.

The most direct evidence comes from the analysis of SOS-constitutive *lexA*(Def) cells (**Fig 6**). Introduction of the *lexA*(Def) mutation increased the concentration of DinB-YPet to nearly 100 nM; more than 15-times higher than the concentration present in undamaged *lexA*^+^ cells. Despite this increase in concentration, there were almost no pol IV foci visible in the *lexA*(Def) cells. This indicates that pol IV concentrations up to 100 nM are insufficient for pol IV to enter the replisome, or for that matter, any other binding site on the DNA. In contrast, pol IV is able to access the DNA in cells treated with DNA damaging agents, even when the pol IV concentration was significantly below 100 nM. Thus, it appears that DNA damage is required for pol IV to access the DNA, at least within the concentration regimes expected to occur in wild-type cells. Interestingly, the pol IV concentration did affect the number of pol IV molecules that bound to each binding site on the DNA. Expression of DinB-eYFP from a low-copy plasmid increased the concentration of labelled pol IV up to 14-fold relative to when DinB-YPet was expressed from the chromosome. This induced only a mild increase in the proportion of pol IV foci that colocalised with replisomes, however the number of molecules present within each focus increased: in the absence of damage there were 1–2 DinB-YPet molecules per focus, increasing to 3–10 molecules per focus when DinB-eYFP was expressed from the plasmid; in the presence of damage there were 3–4 DinB-YPet molecules per focus, increasing to >30 molecules per focus when DinB-eYFP was expressed from the plasmid. Thus, within the bounds of cellular pol IV concentrations, higher pol IV concentrations do not open up new binding sites at replisomes, or any other site on the DNA. High concentrations do, however, allow more pol IV to bind at each binding site.

### Pol IV is granted only temporary access to replisome regions

We conclude that pol IV has very limited access to the region close to replisomes, even after the induction of the SOS response. Access to the replisome region, be it direct access to the replisome or access to ssDNA gaps, is restricted to the first 100 minutes after induction of the SOS response (colocalisation drops after this point), and involves only a small subset of the replisomes and pol IV molecules. What factors could temporarily licence pol IV to enter the area of cells close to replication forks?

In eukaryotes, TLS polymerases are licenced to enter replisomes at least in part through ubiquitination of the PCNA sliding clamp [73]. To our knowledge, pol IV and replisome components are not altered biochemically during the SOS response. Pol IV is currently thought to access replisomes through a series of physical interactions that it forms with the β-sliding clamp and pol III [74–78]. Such interactions could conceivably provide pol IV with access to gaps behind the replisome. These gaps are unlikely to contain pol III HE. There is, however, thought to be an excess of pol III core subunits over pol III HE complexes in *E*. *coli* [77]. Perhaps these free pol III cores compete with pol IV for binding to gaps and the previously described interactions between the two facilitate switching in that context.

It is assumed that when the replisome skips a lesion it leaves a β-sliding clamp behind at the gap. The known interactions of pol IV with the β-sliding clamp are likely to be involved during post-replicative TLS by pol IV. It is difficult to imagine, however, how these interactions could be modulated to provide access to gaps during early stages of the SOS response, while excluding pol IV in late stages of the SOS response. One possibility is that the gaps are no longer created during late stages of the SOS response. Another possibility is that a protein (or complex) binds to either pol IV, or the β-sliding clamp during the late SOS response and prevents pol IV from acting at gaps. Alternatively, a protein (or complex) that is active only during the early stages of SOS could help to recruit pol IV to gaps.

### SOS progresses through periods of distinct enzyme activities

**Fig 7:**
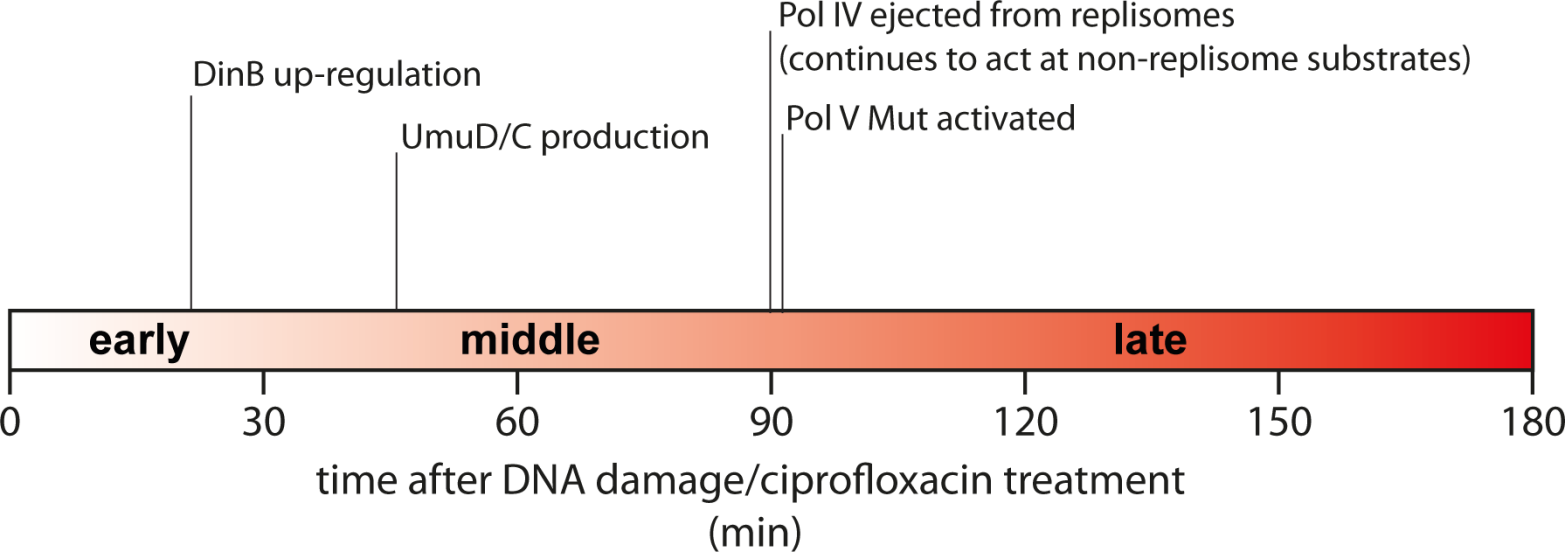
Timeline of translesion DNA synthesis based on single-molecule imaging studies. Pol IV is expressed relatively early after DNA damage is incurred and is allowed access to replisomes until cells abruptly transition into the late stage. At this transition, pol IV is ejected from replisomes and a second TLS polymerase, pol V Mut becomes activated. Pol IV continues to act on non-replisome substrates. The timescale indicated for these transitions is likely to be specific to our growth conditions (EZ glucose medium; APTES-treated flow cell; 37°C). We anticipate that under different conditions the same transitions would be observed, but at different time-points.

A new model of the bacterial SOS-response is emerging in which different proteins are put into play during discrete time periods, as depicted in Fig 7. In the current study, we revealed that pol IV is permitted access to the region close to replisomes 30–100 min after ciprofloxacin addition, after which it is excluded from these regions. This behaviour is not limited to ciprofloxacin treatment: we observe a similar series of events following treatment with both MMS and ultraviolet light (**S10 Fig**). Interestingly, the time-point where pol IV is ejected from the replisome region matches well with the timing of a key event in the regulation of another TLS polymerase, pol V [47]. We previously discovered that pol V becomes activated for TLS 90–120 min after cells are damaged with ultraviolet light. We found that the pol V subunit, UmuC, is produced ~45 min after irradiation. However, the protein is sequestered at the inner membrane, keeping it away from the DNA. From ~90 min after damage, the other critical component of pol V, UmuD′_2_, is produced by RecA*-mediated autoproteolysis of UmuD_2_ [78]. As this point, the pol V complex (UmuD′_2_-UmuC) forms, becomes activated to pol V Mut (UmuD′_2_-UmuC-RecA-ATP) through interaction with a RecA* nucleoprotein filament, and is released from the membrane to catalyse TLS. This same series of events occurs when treating pol V-labelled cells with ciprofloxacin (**S11 Fig**).

That the ejection of pol IV from the replisome region occurs at ~90–100 min, the same time-point at which pol V is released from the membrane (**Fig 7**), suggests a possible functional link between the two enzymes. In the previous study, we demonstrated that pol V Mut does not act at replisomes, ruling out the possibility that pol IV is excluded from replisome regions because it is out-competed by pol V. Based on far-Western blots and pull-down experiments, it has been previously suggested that pol IV interacts with both UmuD_2_ and UmuD′_2_ [78]. UmuD_2_ (and presumably UmuD′_2_) are produced in excess over UmuC, at concentrations similar to pol IV. It is therefore tempting to speculate that UmuD_2_, UmuD′_2_, or both, modulate the access of pol IV to replisome regions. This hypothesis will be tested further in future work. Put together, the results of our previous and current studies suggest that the SOS response progresses through (at least) three stages: an early period (0–30 min) of predominantly error-free repair; a middle period (30–90 min) that includes pol IV-catalysed TLS at gaps behind the replication fork; and finally, a mutagenic period (>90 min) in which pol V Mut is active.

### Experimental procedures

#### Cell constructs and plasmids

A complete list of strains used in the study appears in Table 1. EAW633 is *E*. *coli* K-12 MG1655 *dinB-YPet* [79]. It was made by λ_RED_ recombination [80], replacing the wild-type *dinB* gene with *dinB-YPet* and a mutant FRT-Kanamycin resistance-wt FRT cassette. Positive colonies were selected for kanamycin resistance. The fusion gene *dinB-YPet* encodes pol IV, a C-terminal twenty amino acid spacer (as used in [29]), followed by YPet.

**Table 1:** Strains used in this study.

Strain	Relevant Genotype	Parent strain	Source/technique
MG1655	*dinB*^*+*^ *lexA*^*+*^	-	[79]
EAW18	Δ*dinB*	MG1655	Lambda RED recombination
EAW26	*sulA*^*-*^ *lexA*(Def)	MG1655	[47]
EAW633	*dinB-YPet lexA*^*+*^	MG1655	Lambda RED recombination
**EAW830**	*dinB*(D103N)*-YPet lexA*^*+*^	MG1655	Lambda RED recombination
EAW641	*dinB-YPet dnaQ-mKate2 lexA*^*+*^	EAW633	Lambda RED recombination
EAW643	*dinB-YPet dnaX-mKate2 lexA*^*+*^	EAW633	Lambda RED recombination
**EAW643/pPFB1188**	*dinB-YPet dnaX-mKate2 lexA*^*+*^ *+* pPFB1188 (*dinB-eYFP*)	EAW643	Transformation of EAW643 with pPFB1188 [29]
**EAW192**	*dinB*^*+*^ *dnaQ-mKate2 lexA*^*+*^	MG1655	[48]
**EAW203**	*dnaX-YPet dnaQ-mKate2 dinB*^*+*^ *lexA*^*+*^	JJC5945	[48]
**SSH001**	Δ*dinB dnaQ-mKate2 lexA*^*+*^	EAW192	Transduction of EAW192 with P1 grown on SF2006 [8]
**SSH001/pPFB1188**	Δ*dinB dnaQ-mKate2 lexA*^*+*^ *+* pPFB1188 (*dinB-eYFP*)	SSH001	Transformation of SSH001 with pPFB1188 [29]
RW1588	*dinB-YPet dnaX-mKate2 sulA*::kan^R^	EAW643	Transduction of EAW643 with P1 grown on EAW26
RW1594	*dinB-YPet dnaX-mKate2 sulA*::kan^R^ *lexA*(Def) Cm^R^	RW1588	Transduction of RW1588 with P1 grown on DE406
EAW282	*dnaX-YPet umuC-mKate2 lexA*^+^	JJC5945	[47]
CC108	*dinB*^*+*^; F' plasmid *dinB*^+^	-	[24]
FC1243	Δ*dinB*; F' plasmid Δ*dinB*	CC108	[24]
YG2247	*dinB*^*+*^; F' plasmid Δ*dinB*	CC108	[24]

EAW641 and EAW643 are two-colour strains derived from EAW633. The kanamycin resistance marker in EAW633 was removed via FLP-FRT recombination using the plasmid pLH29 [80]. To construct EAW643, λ_RED_ recombination was used to replace the *dnaX* gene of EAW633 with *dnaX-mKate2* and a mutant FRT-Kanamycin resistance-wt FRT cassette. Colonies were selected for kanamycin resistance. The *dnaX-mKate2* fusion encodes the τ-subunit of pol III HE, a C-terminal 11 amino acid linker followed by mKate2. EAW641 was constructed in a similar manner, replacing the *dnaQ* gene in EAW633 with a *dnaQ-mKate2* fusion.

To increase the intracellular concentration of labelled pol IV, we used the plasmid pPFB1188, which expresses DinB-eYFP (pol IV labelled at its C-terminus with eYFP, through a twenty amino-acid linker; [30]). To generate EAW643 pPFB1188 cells, we transformed EAW643 cells with pPFB1188, selecting for ampicillin resistance. Cells carrying a replisome marker, but lacking *dinB* were used in control measurements. SSH001 is *E*. *coli* MG1655 *dnaQ-mKate2 lexA*^*+*^ *dinB*::kan^R^. It was made by transferring *dinB*::kan^R^ by P1 transduction from SF2006 [8] into EAW192 [48]. SSH001 pPFB1188 was generated by transforming SSH001 cells with pPFB1188 [30].

RW1594 is *E*. *coli* MG1655 *dinB-YPet dnaX-mKate2 lexA*(Def) *sulA*::kan^R^. It was made in two steps: first the wild-type *sulA+* gene of EAW643 was replaced with *sulA*::kan by P1 transduction from EAW26 [47], to create RW1588; then *lexA51*(Def) *malB*::Tn*9* was transferred from DE406 [81] into RW1588 by P1 transduction, selecting for chloramphenicol resistance. To confirm the presence of the *lexA*(Def) genotype, colonies were then screened for high levels of RecA expression by Western blotting with anti-RecA antibodies [82].

#### Western blotting for DinB expression levels

Cell cultures were grown in Luria-Bertani media at 37°C. The following morning, they were diluted 1:100 in fresh media until they reached exponential phase (OD_600_ ~0.5). Where noted, cultures were treated with 30ng/mL ciprofloxacin for 2 hours prior to harvesting. After cells were harvested by centrifugation, the cell pellet was resuspended in NuPage LDS sample buffer (Novex) and freeze-thawed to produce whole cell extracts. Dilutions of purified pol IV protein were made in FC1243 (Δ*dinB*) whole cell extracts. Aliquots of whole cell extracts, representing approximately 1.5 × 10^8^ cells, or DinB dilutions (containing 0.5–8 ng of purified DinB [42]), were electrophoresed in NuPage 4–12% Bis-Tris gels (Novex). Proteins were transferred to an Invitrolon PVDF membrane (Novex) which was probed with a 1:5000 dilution of purified rabbit anti-DinB antibodies (a kind gift from Patricia Foster [30]) and subsequently probed with a 1:5000 dilution of Goat Anti-Rabbit IgG (H+L)-AP Conjugate (BioRad). Using the CDP-Star chemiluminescent assay (Applied Biosystems), the DinB proteins were visualized on Carestream Biomax XAR film after various exposure times.

#### 4-nitroquinolone-1-oxide survival assay

Cells (MG1655, EAW18, and EAW633) were grown in LB overnight at 37°C. The next day, a 1/1000 dilution of each culture was grown to mid log phase (OD_600_ = 0.2), then stored on ice. These cultures were then serially diluted by factors of ten down to 10^−5^. A spot (5 μL) of the OD 0.2 culture and each dilution was plated on an agar plate containing 8 μM NQO. The plate was incubated at 37°C for 18 h.

#### Ciprofloxacin resistance assay

The assay was carried out as described in reference [9]. Cells (MG1655, EAW18, EAW633, EAW643) were grown in LB at 37°C for 25h. For each culture, a 10^−6^ dilution was prepared. 150 ΔL of diluted cells were plated on a LB agar plate and incubated overnight at 37ΔC to count for viable cells. The mutagenesis assay was performed by plating 150 μL of each saturated overnight culture (corresponding to approximately 10^8^ cells) on an LB agar plate containing 40 ng/mL ciprofloxacin. For each strain 5 plates were prepared and incubated at 37°C.

On day one, all colonies of the LB agar plates were counted to determine the number of viable cells that were originally present in each overnight culture. On day 4, colonies on the ciprofloxacin containing plates were counted. These were interpreted as pre-existing mutations [9]. Colonies were counted again on day 8 and 13 and interpreted as resistant colonies formed as a result of mutagenesis induced by ciprofloxacin. The numbers of new colonies appearing between days 4–8 and 8–13 were calculated and normalised against the number of viable cells in each culture. The number of viable cells as a function of time was determined using the count taken at day 1 and loss-of-viability rates measured previously [9].

#### Fluorescence microscopy

Wide-field fluorescence imaging was performed on an inverted microscope (IX-81, Olympus with a 1.49 NA 100x objective) in an epifluorescence configuration, as described previously [47]. Continuous excitation is provided using semidiode lasers (Sapphire LP, Coherent) of the wavelength 514 nm (150 mW max. output) and 568 nm (200 mW max. output). DnaX-mKate2 and DnaQ-mKate2 were imaged using yellow excitation light (λ = 568 nm) at high intensity (2750 Wcm^-2^), collecting emitted light between 610–680 nm (ET 645/75m filter, Chroma) on a 512 × 512 pixel EM-CCD camera (C9100-13, Hamamatsu). For DinB-YPet and DinB-eYFP imaging, we used green excitation (λ = 514 nm) at lower power (160 Wcm^-2^) for DinB-YPet strains (EAW641 and EAW 643) and 60 Wcm^-2^ for the DinB-YPet+DinB-eYFP strain EAW643 pPFB1188, collecting light emitted between 525–555 nm (ET540/30m filter, Chroma).

Rapid acquisitions (movies of 300 × 34 ms frames, continuous excitation with 514 nm light) were collected to characterise the motions of DinB-YPet and DinBD103N-YPet molecules, and to determine the number of DinB-YPet molecules per cell. Time-lapse movies were recorded to visualise changes in DinB-YPet expression and measure colocalisation with replisome markers. For EAW641 and EAW643 cells, sets of three images were recorded (bright-field [34 ms exposure], YPet fluorescence [50 ms exposure]; mKate2 fluorescence [100 ms exposure]) at an interval of 5 min for 3h. All images were analysed with ImageJ [65].

#### Flow cell designs

All imaging was carried out on cultures growing in home-built flow cells. Most imaging was carried out in quartz-based flow cells, similar to those used in our previous study [47]. These flow cells were assembled from a no. 1.5 coverslip (Marienfeld, REF 0102222), a quartz top piece (45x20x1 mm) and PE-60 tubing (Instech Laboratories, Inc.). Prior to flow-cell assembly, coverslips were silanized with aminopropyltriethoxy silane (Sigma Aldrich, Alfa Aeser). First, coverslips were sonicated for 30 min in a 5M KOH solution to clean and activate the surface. The cleaned coverslips were rinsed thoroughly with MilliQ water, then treated with a 5% (v/v) solution of amino-propyl-triethoxysilane in MilliQ water. The coverslips were subsequently rinsed with ethanol and sonicated in ethanol for 20 seconds. Afterwards, the coverslips were rinsed with MilliQ water and dried in a jet of N_2_. Silanised slides were stored under vacuum prior to use.

To assemble each flow cell, polyethylene tubing (BTPE-60, Instech Laboratories, Inc.) was glued (BONDiT B-482, Reltek LLC) into two holes that were drilled into a quartz piece. After the glue solidified overnight, double-sided adhesive tape was stuck on two opposite sides of the quartz piece to create a channel. Then, the quartz piece was stuck to an APTES-treated coverslip. The edges were sealed with epoxy glue (5 Minute Epoxy, DEVCON home). Each flow cell was stored in a desiccator under mild vacuum while the glue dried. Typical channel dimensions were 45 mm × 5 mm × 0.1 mm (length × width × height).

Data shown in **Figs 2 and 3** were collected in a three-channel PDMS-based flow cell. A commercial PDMS kit (Dow Corning, SYLGARD 184 silicone elastomer kit) was used to obtain a 10:1 (polymer:curing agent) mixture. The mixed resin was poured in an aluminium mold that has three ridges, creating PDMS blocks with channel dimensions (0.1 mm, 0.5 mm and 1.9 mm). After pouring, the polymer was allowed to solidify at 65°C overnight. The next day, 1 mm holes were punched in the PDMS block for the in- and outlet tubing. Then, the PDMS block was covalently attached to a clean glass coverslip (KOH treated as above) by plasma treatment. After plasma bonding, PE60 tubing was pushed into each hole. As a final step, the flow cell surface was silanised by pulling 5% (v/v in water) amino propyl triethoxy silane solution through the channel with a syringe. The silanization reaction was allowed to proceed for 15 min before the channels were flushed with MilliQ water.

#### Imaging in flow cells

For all imaging experiments, cells were grown at 37°C in EZ rich defined medium (Teknova) that contained 0.2% (w/v) glucose. EAW633, EAW641 and EAW643 cells were grown in the presence of kanamycin (25 μg/mL), EAW643 pPFB1188 was grown in the presence of ampicillin (100 μg/mL) and RW1594 was grown in the presence of chloramphenicol (25 μg/mL). Cells were loaded into flow cells, allowed a few minutes to associate with the APTES surface, then loosely associated cells were removed by pulling through fresh medium. The experiment was then initiated by either changing the input solution to medium containing 30 ng/mL ciprofloxacin or 0.2 ng/ml MMS, or by irradiating cells *in situ* with 254 nm UV light from a mercury lamp (UVP) at a fluence of 30 J.m^-2^. In each case, medium was pulled through the flow cell throughout the measurement using a syringe pump, at a rate of 50 μL/min.

#### Analysis of pol IV upregulation

We selected regions of images occupied by cells to obtain information about pol IV upregulation upon ciprofloxacin treatment (>200 cells; all 5 min frames during the 3h experiment). MicrobeTracker 0.937 [83], a MATLAB script, was used to create cell outlines as regions of interest (ROI). We manually curated cell outlines designated by MicrobeTracker to ensure accuracy and to ensure that only non-overlapping, in-focus cells were selected for analysis. These ROI were imported in ImageJ 1.50i [84]. The cell outlines were then used to measure mean cell intensities, cell lengths and the number of foci per cell. Parameters describing foci (number, positions and intensities) were obtained using a Peak Fitter plug-in, described previously [47].

#### Analysis of colocalisation events of pol IV with replisomes

Foci were classed as colocalised if their centroid positions (determined using our peak fitter tool) fell within 2 px (200 nm) of each other. We determined that for DinB-YPet–DnaX-mKate2 localisation the background of pol IV foci expected to colocalise with replisomes purely by chance is ~4%. This was calculated by taking the area of each cell occupied by replisome foci (including the colocalisation search radius) and dividing by the total area of the cell. The value of 4% corresponds to the mean of measurements made over >300 cells. As the number of pol IV foci changes in time, the proportion of replisome foci expected to colocalise with pol IV foci by chance also changes in time. At the beginning of the measurement, there are almost zero pol IV foci, thus there is close to zero chance that a replisome focus will colocalise with a pol IV focus. At t = 30 min, chance colocalisation is expected to be 5% and at t = 120 min, the chance for co-localisation 3%.

#### Analysis of pol IV copy numbers per cell

The number of pol IV molecules per cell and thus the intracellular concentration is extracted from the change in integrated intensity under each cell outline during rapid acquisition photobleaching measurements, as described previously [47]. The intensity decay for each cell includes contributions not just from YPet bleaching, but also from cellular auto-fluorescence and background signals from the flow cell surface. To obtain a background-free measure of the YPet photobleaching rate, we measured the number of foci detected over hundreds of cells during bleaching. The number of foci over time followed a single exponential decay with τ = 6 s. Returning to the integrated cell intensity decays, we found that signals followed a two-exponential decay, with τ_1_ = 6 s and τ_2_ ≈ 60 s. Wild-type cells, expressing no YPet, gave single-exponential decays with τ ≈ 60 s, indicating that the τ = 6 s decay seen for YPet-expressing cells arises purely due to YPet bleaching. It was therefore possible to easily extract the YPet intensity from the slower decaying auto-fluorescence and background by fitting with a two-exponential function.

First, the images were corrected for the electronic offset and flattened to correct for inhomogeneity of the excitation beam. We then fit the cellular intensity decay with a two exponential function f(x), fixing τ_1_ to 6 s^1^:

$$f\left(x\right)=A_{1}\cdot exp\left(‑x/\tau_{1}\right)+A_{2}\cdot \exp\left(‑x/\tau_{2}\right).$$

For each cell, the amplitude A_1_ is an accurate measure of the mean YPet signal per pixel. Multiplying by the cell area gives the integrated YPet intensity, which was used to determine the number of YPet molecules per cell.

The mean intensity of individual YPet molecules was determined by analysing single-molecule return events (see **S1 Fig**). For each cell, the number of DinB-YPet molecules was then calculated by dividing the integrated YPet intensity, measured by two-exponential fitting of cell-area decays, by the mean single-molecule intensity. The concentration was calculated using the volume of each cell, determined during cell outline assignation in MicrobeTracker.

## Supporting information

S1 Fig Measurement of DinB and DinB-YPet molecules per cell in different backgrounds. Western blots were developed using anti-DinB antibodies. In addition to DinB-specific bands, a series of bands for cross-reacting species were observed. The slowest migrating of these was used as an internal reference for the amount of cell extract loaded in each lane. (A) Calibration for DinB loading. Lanes: i) molecular weight marker, ii) 8 ng DinB, iii) 4 ng DinB, iv) 2 ng DinB, v) 1 ng DinB, vi) 0.5 ng DinB, vii) molecular weight marker. (B) Corresponding calibration plot: band intensity is plotted against loaded DinB (ng). Lanes with 0.5 ng, 1 ng and 2 ng DinB were included in calibration plot, 4 ng and 8 ng were excluded due to saturation. The intensities plotted for each band are the integrated intensity of the DinB band divided by the integrated intensity of the reference band. Amounts of DinB present in cell extracts (C–F) were calculated from a line of best fit (y = 5.7773x; R^2^ = 0.72553). (C) Western blot of extracts from untreated cells. Lanes: i) molecular weight marker, ii) FC1243 (*ΔdinB*), iii) MG1655 (*dinB*^*+*^), iv) EAW633 (*dinB-YPet*), v) YG2247 (*dinB*^*+*^; F' plasmid - *ΔdinB*), vi) CC108 (*dinB*^*+*^; F' plasmid—*dinB*^*+*^), vii) molecular weight marker. Bands corresponding to full length DinB-YPet are clearly visible in lane iv. A small amount of two DinB-containing fragments are also visible. Fragment 1 corresponds to DinB+linker. Fragment 2 corresponds to DinB +/- one or two residues. (D) Calculated molecules per cell for untreated cells (Western blot, panel C). Total DinB levels in EAW633 (DinB-YPet) cells are similar to wild-type DinB levels, although ~25% is proteolysed within the cells. YG2247 have DinB at equivalent levels to MG1655, whereas, CC108 have tenfold higher levels than MG1655. This is due to the fact that CC108 cells contain the F' plasmid, which provides a second copy of *dinB*. Levels in CC108 may be somewhat underestimated due to saturation of DinB bands. (E) Two Western blots of extracts from cells. Lanes from left Western blot: i) molecular weight marker, ii) FC1243 (*ΔdinB*) untreated, iii) FC1243 (*ΔdinB*) ciprofloxacin-treated, iv) MG1655 (*dinB*^*+*^) untreated, v) MG1655 (*dinB*^*+*^) ciprofloxacin-treated, vi) EAW633 (*dinB-YPet*) untreated, vii) EAW633 (*dinB-YPet*) ciprofloxacin-treated, viii) molecular weight marker. Bands corresponding to full length DinB-YPet are clearly visible in lane vi-vii. A small amount of two DinB-containing fragments are also visible. Lanes from right Western blot: i) molecular weight marker, ii) YG2247 (*dinB*^*+*^) untreated, iii) YG2247 (*dinB*^*+*^; F' plasmid - *ΔdinB*) ciprofloxacin-treated, iv) CC108 (*dinB*^*+*^; F' plasmid—*dinB*^*+*^) untreated, v) CC108 (*dinB*^*+*^) ciprofloxacin-treated, vi) molecular weight marker. (F) Calculated molecules per cell for untreated and ciprofloxacin-treated cells (two Western blots, panel E). In untreated cells, DinB-YPet is expressed at levels equivalent to wild-type DinB, however ~58% is proteolysed within the cells. In comparison to MG1655, YG2247 expresses similar levels of DinB, whereas, CC108 have tenfold higher expression levels. The band for CC108 is saturated, however, and thus likely to be underestimated. In ciprofloxacin-treated EAW633 (DinB-YPet) cells, levels are similar to wild-type DinB levels, however ~31% is proteolysed within the cells. Comparing to MG1655, YG2247 expressed ~1.7 fold more DinB, whereas, CC108 have fourfold higher expression levels. This however might be an underestimate due to the saturated band.
(TIF)

S2 Fig Measurement of the number of DinB-YPet molecules per focus by analysis of photobleaching trajctories. (A) Representative photobleaching trajectory showing bleaching of a single DinB-YPet molecule. For each focus, the intensity within a 5 × 5 pixel selection box was monitored as a function of time as foci photobleached. Each measurement was locally background-corrected by subtracting the mean intensity within a 2 pixel-wide ring outside each focus. The red line indicates a fit of intensity levels derived from change-point analysis [47]. (B) Histogram of single-molecule intensities. Once the majority of DinB-YPet in cells had photobleached, foci occasionally appeared as individual molecules returned to the bright (fluorescent) state. These foci were fit with 2D Gaussian functions to determine the integrated fluorescence intensities. The measured intensities were narrowly distributed, with a mean value of 1850 arbitrary units. This value represents the mean intensity of a single DinB-YPet molecule. (C–D) Histograms of intensities for DinB-YPet foci, 30 min (C) and 100 min (D) after addition of ciprofloxacin. The initial intensities of foci were determined from photobleaching trajectories using change-point analysis [47]. This value was then divided by the single-molecule intensity 1850 to obtain the number of molecules present in each focus.
(TIF)

S3 Fig Comparison of pol IV-replisome colocalisation in EAW641 and EAW643. Foci located within 200 nm of each other were defined as being colocalised. (A) Graph indicating the proportion of pol IV foci that contain a colocalised replisome focus in EAW641 cells (red line) and EAW643 cells (black line). (B) Graph indicating the proportion of replisome foci that contain a colocalised pol IV focus in EAW641 cells (red line) and EAW643 cells (black line). Shaded areas (A–B) indicate the standard error of the proportion. The total number of cells analysed were not determined in these measurements. We conservatively estimate that >300 cells were used in each measurement. The DnaX-mKate2 dataset includes a total of 17005 DnaX-mKate2 foci and 12408 DinB-YPet foci. The DnaQ-mKate2 dataset includes 7451 DnaQ-mKate2 foci and 3166 DinB-YPet foci.
(TIF)

S4 Fig Colocalisation measurements for images of EAW643 cells recorded with a longer (300 ms) exposure time. Foci located within 200 nm of each other were defined as being colocalised. (A) Plot of the number of pol IV and replisome foci per EAW643 cell as a function of time. Some cells were lost from the coverslip surface during the measurement. A total of 134 cells remained bound and were analysed over the full course of the measurement. (B) Graph indicating the proportion of replisome foci that contain a colocalised pol IV focus. (C) Graph indicating the proportion of pol IV foci that contain a colocalised replisome focus. Error bars (B–C) indicate the standard error of the proportion. The total number of cells analysed were not determined in these measurements. We conservatively estimate that >300 cells were used in each measurement. The analysis includes a total of 7160 DnaX-mKate2 foci and 1027 DinB-YPet foci.
(TIF)

S5 Fig Pol IV behaviour in cells expressing both DinB-YPet (from the *dinB* locus on the chromosome) and DinB-eYFP (from the plasmid pPFB1188). (A) Representative microscope images comparing yellow fluorescent protein signals in EAW643 (DinB-YPet only; top row) and EAW643 pPFB1188 (DinB-YPet + DinB-eYFP; bottom row) cells, 100 min after ciprofloxacin addition. The left and right columns contain the same images, but with different intensity ranges displayed. (B) Photobleaching trajectories for DinB foci in EAW643 pPFB1188 (DinB-YPet + DinB-eYFP) cells. Trajectories were measured as illustrated in S1 Fig. Derivation of the intensity of a single YPet molecule (1850 arbitrary units) is shown in S1B Fig. The intensity of a single eYFP molecule (1200 arbitrary units) was estimated based on the relative extinction coefficients and quantum yields of YPet and eYFP [85].
(TIF)

S6 Fig Comparison of pol IV-replisome colocalisation in cells expressing labelled pol IV from the chromosome (DinB-YPet), a plasmid (DinB-eYFP), or both. Foci located within 200 nm of each other were defined as being colocalised. Measurements were made on cells treated with 30 ng/ml ciprofloxacin for 60 min in the context of a flow cell. (A) Bar graph indicating the proportion of pol IV foci that contain a colocalised replisome focus. (B) Bar graph indicating the proportion of replisome foci that contain a colocalised pol IV focus. Bar colours (A–B) indicate cell type: EAW643 (blue), EAW643 pPFB1188 (red), EAW641 (green) and SSH001 pPFB1188 (yellow). Error bars indicate the standard error of the proportion. The total number of cells analysed were not determined in these measurements. We conservatively estimate that >300 cells were used in each measurement. The DnaX-mKate2 DinB-YPet dataset includes a total of 1178 DnaX-mKate2 foci and 907 DinB-YPet foci. The DnaX-mKate2 DinB-YPet + DinB-eYFP dataset includes 1165 DnaX-mKate2 foci and 1264 DinB-YPet/DinB-eYFP foci. The DnaQ-mKate2 DinB-YPet dataset includes a total of 739 DnaQ-mKate2 foci and 413 DinB-YPet foci. The DnaQ-mKate2 DinB-YPet + DinB-eYFP dataset includes 386 DnaQ-mKate2 foci and 280 DinB-YPet/DinB-eYFP foci.
(TIF)

S7 Fig Increased rates of lysis in cells expressing DinB-eYFP from pPFB1188. (A) Representative bright-field images of EAW643 cells (top two panels) and SSH001 pPFB1188 cells (bottom two panels), 180 min after the addition of 30 ng/ml ciprofloxacin. Arrows indicate the positions of cells that have lysed. (B) Bar graph showing the percentage of cells that lyse by the 180 min time-point for MG1655, EAW643 and SSH001 pPFB1188 cells. The number of cells that were tracked were as follows: MG1655, 102 cells; EAW643, 132 cells; SSH001 pPFB1188, 232 cells.
(TIF)

S8 Fig Intensity vs time trajectories for DinB-YPet signals in the vicinity of replisomes in ciprofloxacin-treated EAW643 cells. 5×5 pixel regions of interest were placed at replisome foci, then used to monitor fluctuations in DinB-YPet signals (see Fig 6A). A subset of 42 trajectories were selected randomly from a total of 470 trajectories. To allow comparison with DinB-YPet singles in *lexA*(Def) cells, where expression levels are too high to observe single-molecule foci, we present only a portion of each trajectory, starting at a time-point (150 ms) where ~50% of DinB-YPet molecules have already photobleached. In ciprofloxacin-treated EAW643 cells, DinB-YPet signals are frequently elevated in the vicinity of replisomes for multiple frames, indicating long-lived binding events.
(TIF)

S9 Fig Intensity vs time trajectories for DinB-YPet signals in the vicinity of replisomes in untreated *lexA*(Def) cells. 5×5 pixel regions of interest were placed at replisome foci, then used to monitor fluctuations in DinB-YPet signals (see Fig 6A). A subset of 42 trajectories were selected randomly from a total of 65 trajectories. A portion of each trajectory is presented, starting at a time-point (150 ms) where ~50% of DinB-YPet molecules have already photobleached, allowing single-molecule foci to be observed. In untreated *lexA*(Def) cells few events are visible in which the DinB-YPet is elevated in the vicinity of replisomes for more than a single 34 ms frame, indicating few long-lived binding events.
(TIF)

S10 Fig Comparison of pol IV-replisome colocalisation in cells treated with different DNA-damaging agents: (A) 30 ng/ml ciprofloxacin, (B) 0.26 ng/ml methyl methanesulfonate, (C) ultraviolet light (fluence = 30 J/m^2^, flux density = 3.3 W/m^2^, λ = 254 nm). Colocalisation (A–C) was measured in two ways: the proportion of DinB-YPet foci that contain a colocalised DnaX-mKate2 (replisome) focus, and the proportion of DnaX-mKate2 foci that contain a colocalised DinB-YPet focus. Shaded areas indicate the standard error of the proportion. All DNA-damaging agents produce a distinctive drop in colocalisation 90–120 min after treatment. We conservatively estimate that >200 cells were used in each measurement.
(TIF)

S11 Fig Time-lapse imaging of pol V-labelled EAW282 (*dnaX-YPet umuC-mKate2*) cells following treatment with 40 ng/ml ciprofloxacin. We previously discovered that pol V is spatially regulated: the UmuC protein accumulates at the cell membrane, until the active form pol V Mut (UmuD′_2_-UmuC-RecA-ATP) is formed and released into the cytosol [47]. This was monitored by observing the change in cellular localisation of UmuC-mKate2 as a function of time. When cells are instead treated with ciprofloxacin, UmuC-mKate2 goes through a very similar progression of localisation states. In the example shown in this figure, UmuC-mKate2 is initially absent (0–1 h), then membrane associated (1.5–2 h), then cytosolic (2.5–3 h). The timing of these transitions varies from cell to cell. Typically, no UmuC-mKate2 is visible until 45–90 min after ciprofloxacin treatment. From 45–150 min, UmuC-mKate2 is membrane-associated. UmuC-mKate2 typically remains membrane-associated for approximately 30 min before being released into the cytosol.
(TIF)

S1 Movie Time-lapse imaging of pol IV up-regulation in response to ciprofloxacin treatment. Cells were initially grown in rich medium in the absence of exogenous DNA damage. At t = 0 min, the flow cell inlet was switched to medium containing 30 ng/ml ciprofloxacin. At each field-of-view, a bright-field image and a DinB-YPet fluorescence image were collected every 5 min for 180 min. Time stamp indicates hours after ciprofloxacin addition.
(MP4)
