# Supplementary figures and images for: DNA polymerase IV primarily operates outside of DNA replication forks in Escherichia coli
Source: PLoS Genet. 2018 Jan 19;14(1):e1007161. doi: 10.1371/journal.pgen.1007161 (PMC5792023; doi:10.1371/journal.pgen.1007161)

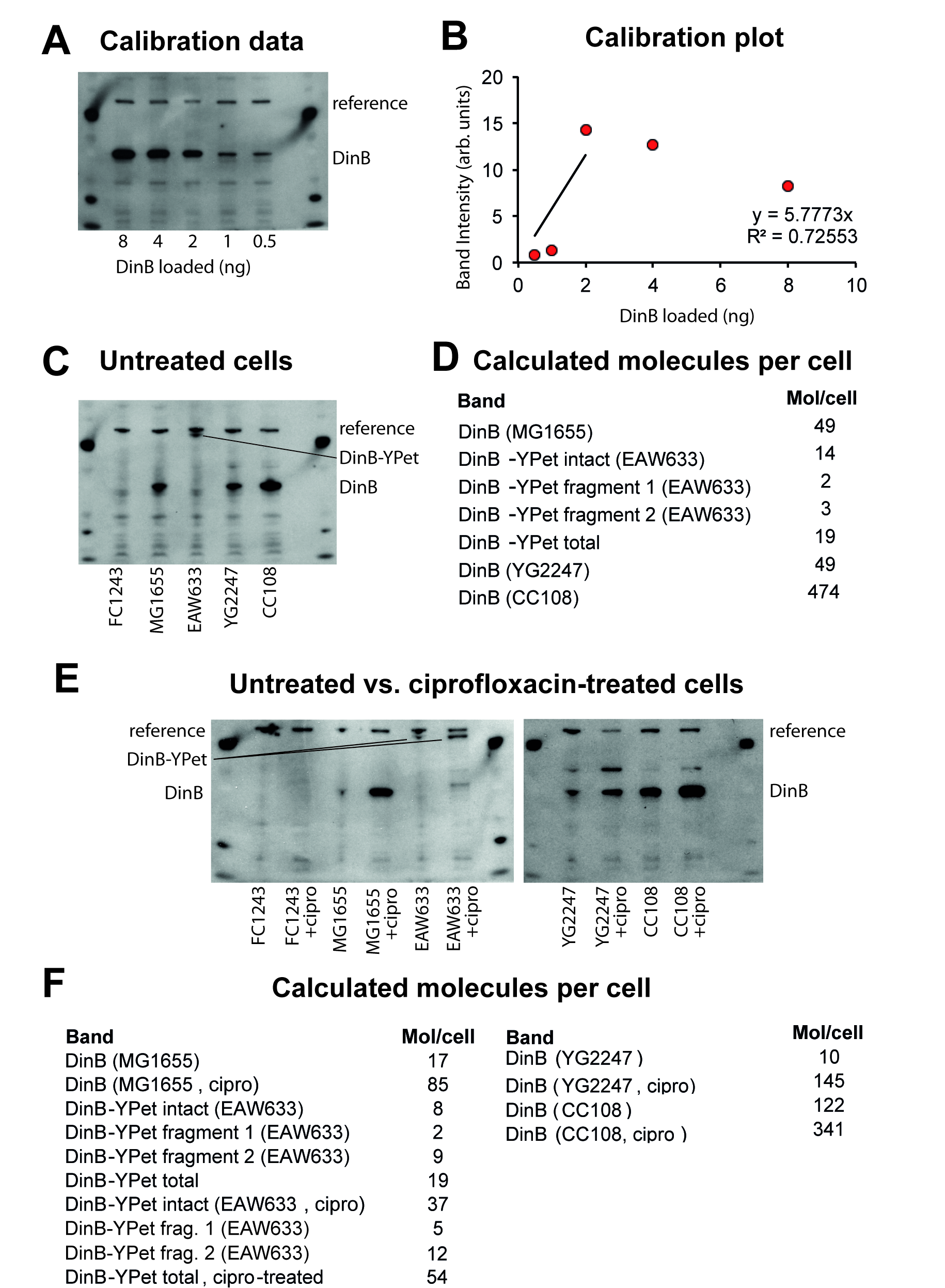

Supplement: S1 Fig — Western blots were developed using anti-DinB antibodies. In addition to DinB-specific bands, a series of bands for cross-reacting species were observed. The slowest migrating of these was used as an internal reference for the amount of cell extract loaded in each lane. (A) Calibration for DinB loading. Lanes: i) molecular weight marker, ii) 8 ng DinB, iii) 4 ng DinB, iv) 2 ng DinB, v) 1 ng DinB, vi) 0.5 ng DinB, vii) molecular weight marker. (B) Corresponding calibration plot: band intensity is plotted against loaded DinB (ng). Lanes with 0.5 ng, 1 ng and 2 ng DinB were included in calibration plot, 4 ng and 8 ng were excluded due to saturation. The intensities plotted for each band are the integrated intensity of the DinB band divided by the integrated intensity of the reference band. Amounts of DinB present in cell extracts (C–F) were calculated from a line of best fit (y = 5.7773x; R2 = 0.72553). (C) Western blot of extracts from untreated cells. Lanes: i) molecular weight marker, ii) FC1243 (ΔdinB), iii) MG1655 (dinB+), iv) EAW633 (dinB-YPet), v) YG2247 (dinB+; F' plasmid - ΔdinB), vi) CC108 (dinB+; F' plasmid—dinB+), vii) molecular weight marker. Bands corresponding to full length DinB-YPet are clearly visible in lane iv. A small amount of two DinB-containing fragments are also visible. Fragment 1 corresponds to DinB+linker. Fragment 2 corresponds to DinB +/- one or two residues. (D) Calculated molecules per cell for untreated cells (Western blot, panel C). Total DinB levels in EAW633 (DinB-YPet) cells are similar to wild-type DinB levels, although ~25% is proteolysed within the cells. YG2247 have DinB at equivalent levels to MG1655, whereas, CC108 have tenfold higher levels than MG1655. This is due to the fact that CC108 cells contain the F' plasmid, which provides a second copy of dinB. Levels in CC108 may be somewhat underestimated due to saturation of DinB bands. (E) Two Western blots of extracts from cells. Lanes from left Western blot: i) molecular weight [file pgen.1007161.s001.tif]

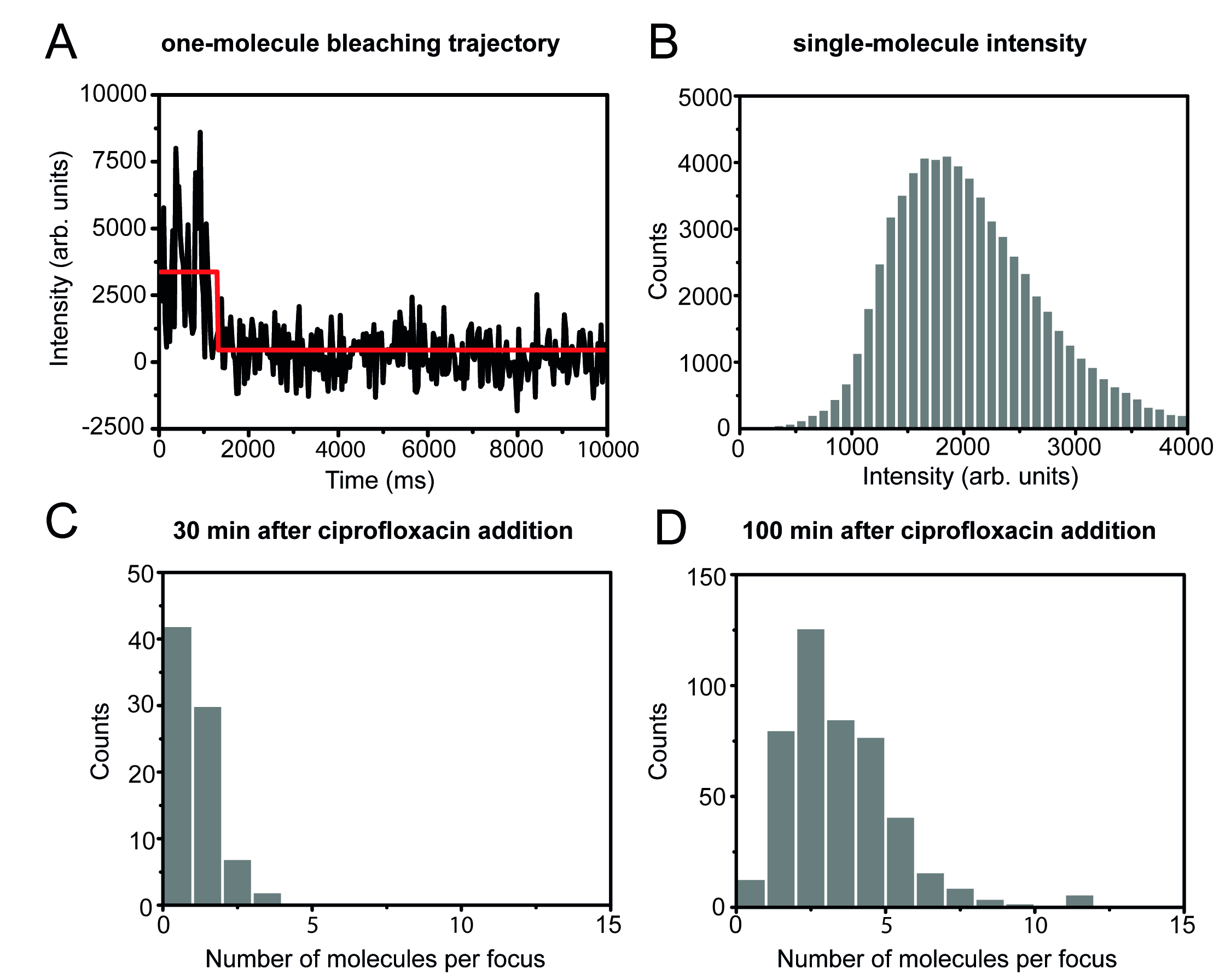

Supplement: S2 Fig — (A) Representative photobleaching trajectory showing bleaching of a single DinB-YPet molecule. For each focus, the intensity within a 5 × 5 pixel selection box was monitored as a function of time as foci photobleached. Each measurement was locally background-corrected by subtracting the mean intensity within a 2 pixel-wide ring outside each focus. The red line indicates a fit of intensity levels derived from change-point analysis [47]. (B) Histogram of single-molecule intensities. Once the majority of DinB-YPet in cells had photobleached, foci occasionally appeared as individual molecules returned to the bright (fluorescent) state. These foci were fit with 2D Gaussian functions to determine the integrated fluorescence intensities. The measured intensities were narrowly distributed, with a mean value of 1850 arbitrary units. This value represents the mean intensity of a single DinB-YPet molecule. (C–D) Histograms of intensities for DinB-YPet foci, 30 min (C) and 100 min (D) after addition of ciprofloxacin. The initial intensities of foci were determined from photobleaching trajectories using change-point analysis [47]. This value was then divided by the single-molecule intensity 1850 to obtain the number of molecules present in each focus. (TIF) [file pgen.1007161.s002.tif]

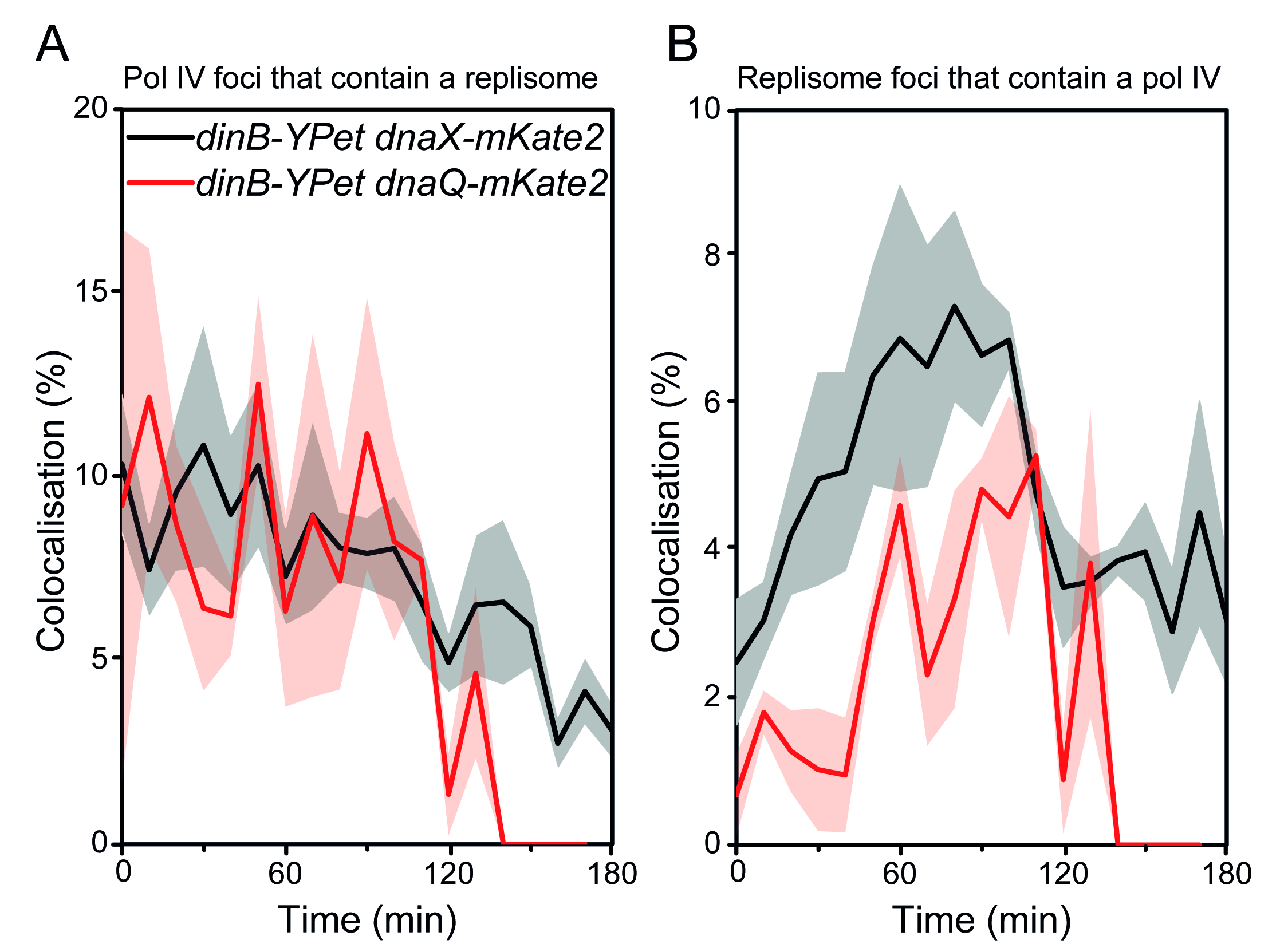

Supplement: S3 Fig — Foci located within 200 nm of each other were defined as being colocalised. (A) Graph indicating the proportion of pol IV foci that contain a colocalised replisome focus in EAW641 cells (red line) and EAW643 cells (black line). (B) Graph indicating the proportion of replisome foci that contain a colocalised pol IV focus in EAW641 cells (red line) and EAW643 cells (black line). Shaded areas (A–B) indicate the standard error of the proportion. The total number of cells analysed were not determined in these measurements. We conservatively estimate that >300 cells were used in each measurement. The DnaX-mKate2 dataset includes a total of 17005 DnaX-mKate2 foci and 12408 DinB-YPet foci. The DnaQ-mKate2 dataset includes 7451 DnaQ-mKate2 foci and 3166 DinB-YPet foci. (TIF) [file pgen.1007161.s003.tif]

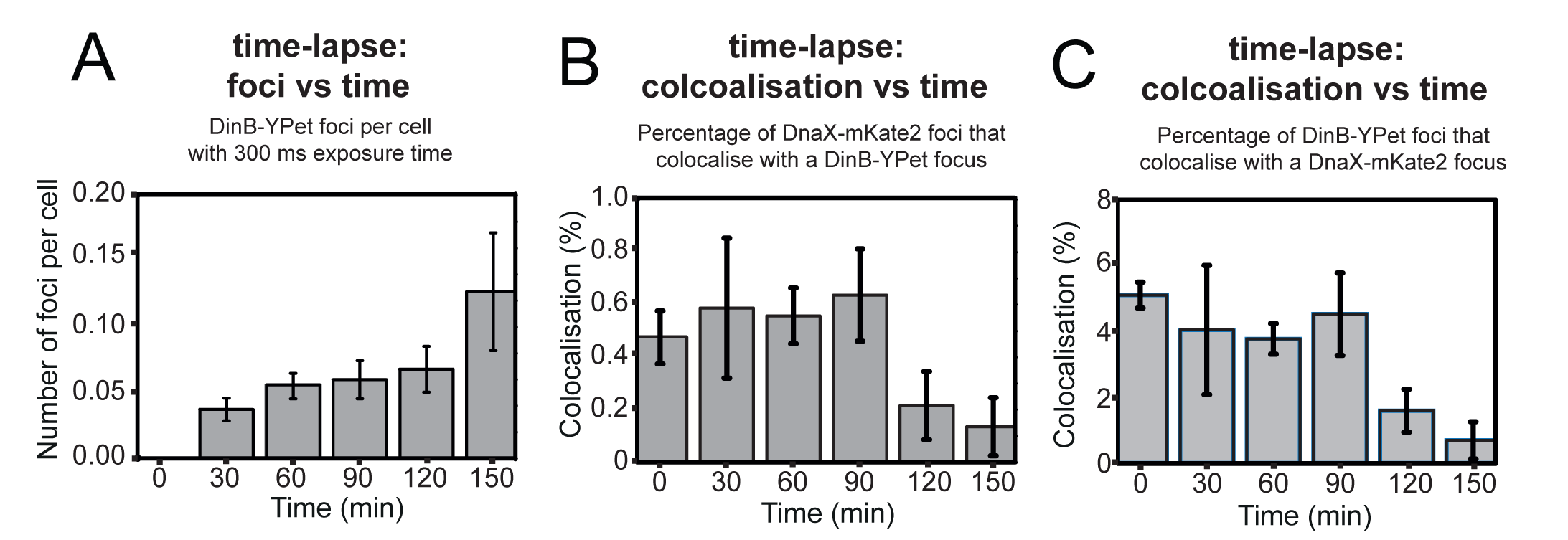

Supplement: S4 Fig — Foci located within 200 nm of each other were defined as being colocalised. (A) Plot of the number of pol IV and replisome foci per EAW643 cell as a function of time. Some cells were lost from the coverslip surface during the measurement. A total of 134 cells remained bound and were analysed over the full course of the measurement. (B) Graph indicating the proportion of replisome foci that contain a colocalised pol IV focus. (C) Graph indicating the proportion of pol IV foci that contain a colocalised replisome focus. Error bars (B–C) indicate the standard error of the proportion. The total number of cells analysed were not determined in these measurements. We conservatively estimate that >300 cells were used in each measurement. The analysis includes a total of 7160 DnaX-mKate2 foci and 1027 DinB-YPet foci. (TIF) [file pgen.1007161.s004.tif]

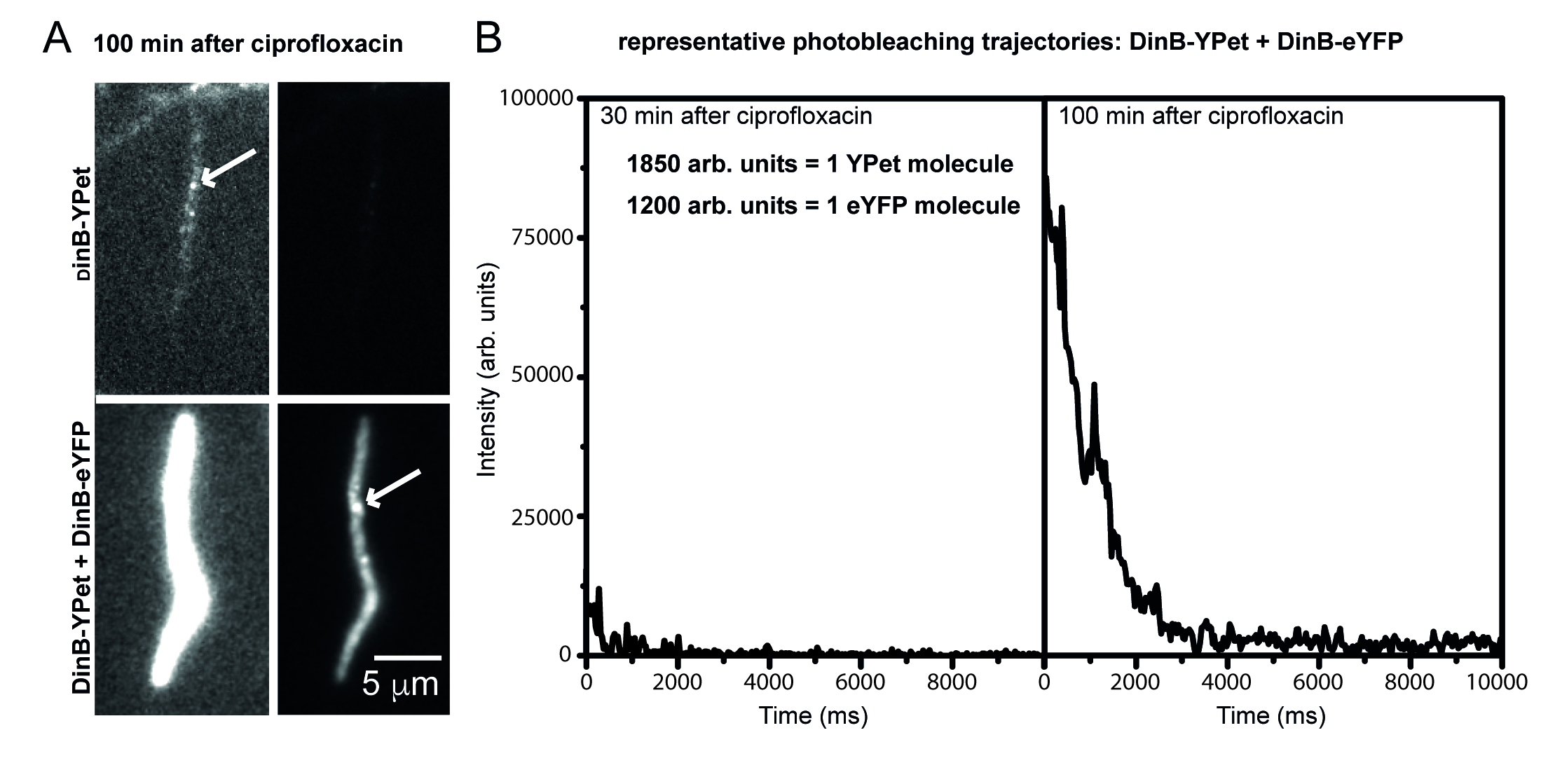

Supplement: S5 Fig — (A) Representative microscope images comparing yellow fluorescent protein signals in EAW643 (DinB-YPet only; top row) and EAW643 pPFB1188 (DinB-YPet + DinB-eYFP; bottom row) cells, 100 min after ciprofloxacin addition. The left and right columns contain the same images, but with different intensity ranges displayed. (B) Photobleaching trajectories for DinB foci in EAW643 pPFB1188 (DinB-YPet + DinB-eYFP) cells. Trajectories were measured as illustrated in S1 Fig. Derivation of the intensity of a single YPet molecule (1850 arbitrary units) is shown in S1B Fig. The intensity of a single eYFP molecule (1200 arbitrary units) was estimated based on the relative extinction coefficients and quantum yields of YPet and eYFP [85]. (TIF) [file pgen.1007161.s005.tif]

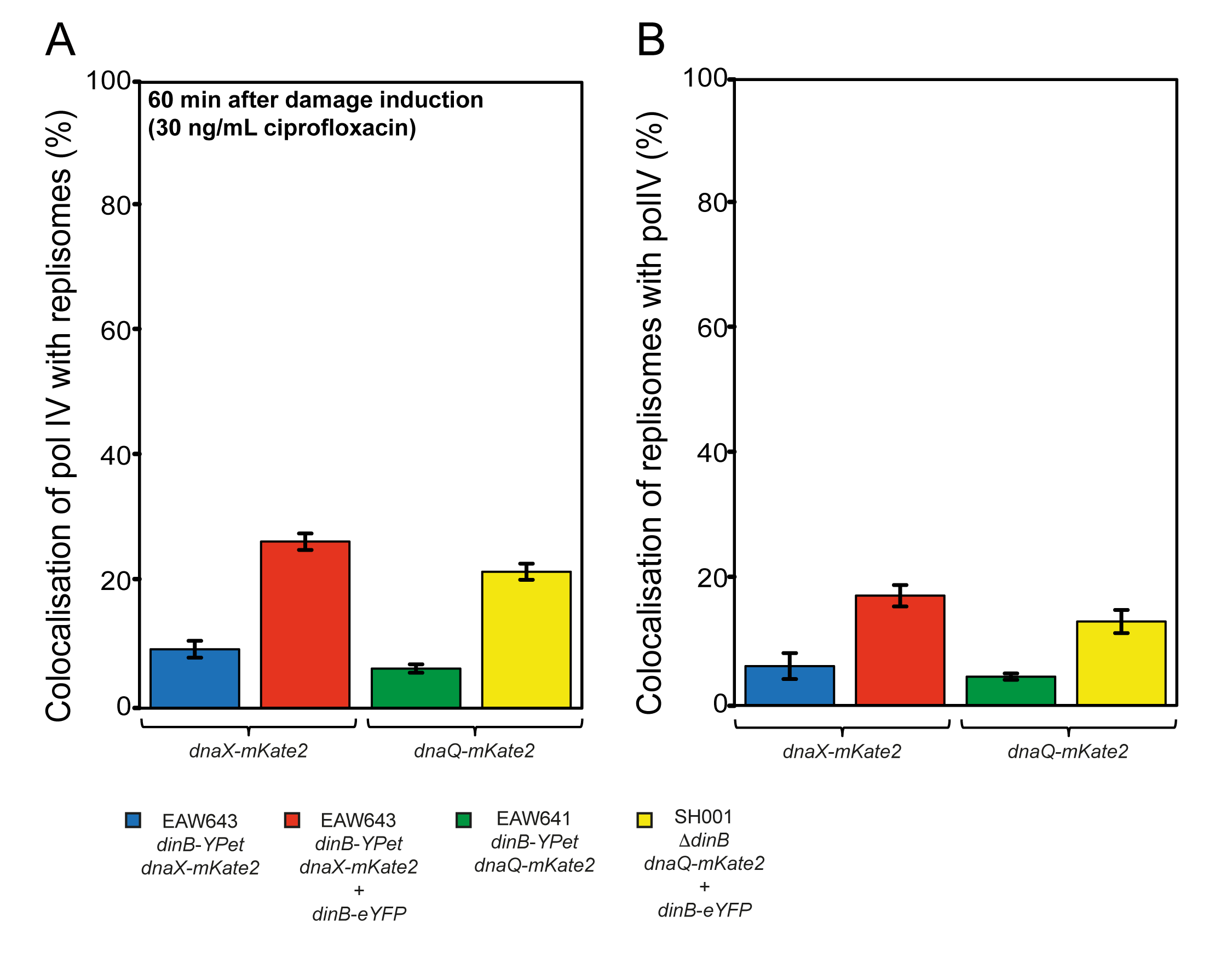

Supplement: S6 Fig — Foci located within 200 nm of each other were defined as being colocalised. Measurements were made on cells treated with 30 ng/ml ciprofloxacin for 60 min in the context of a flow cell. (A) Bar graph indicating the proportion of pol IV foci that contain a colocalised replisome focus. (B) Bar graph indicating the proportion of replisome foci that contain a colocalised pol IV focus. Bar colours (A–B) indicate cell type: EAW643 (blue), EAW643 pPFB1188 (red), EAW641 (green) and SSH001 pPFB1188 (yellow). Error bars indicate the standard error of the proportion. The total number of cells analysed were not determined in these measurements. We conservatively estimate that >300 cells were used in each measurement. The DnaX-mKate2 DinB-YPet dataset includes a total of 1178 DnaX-mKate2 foci and 907 DinB-YPet foci. The DnaX-mKate2 DinB-YPet + DinB-eYFP dataset includes 1165 DnaX-mKate2 foci and 1264 DinB-YPet/DinB-eYFP foci. The DnaQ-mKate2 DinB-YPet dataset includes a total of 739 DnaQ-mKate2 foci and 413 DinB-YPet foci. The DnaQ-mKate2 DinB-YPet + DinB-eYFP dataset includes 386 DnaQ-mKate2 foci and 280 DinB-YPet/DinB-eYFP foci. (TIF) [file pgen.1007161.s006.tif]

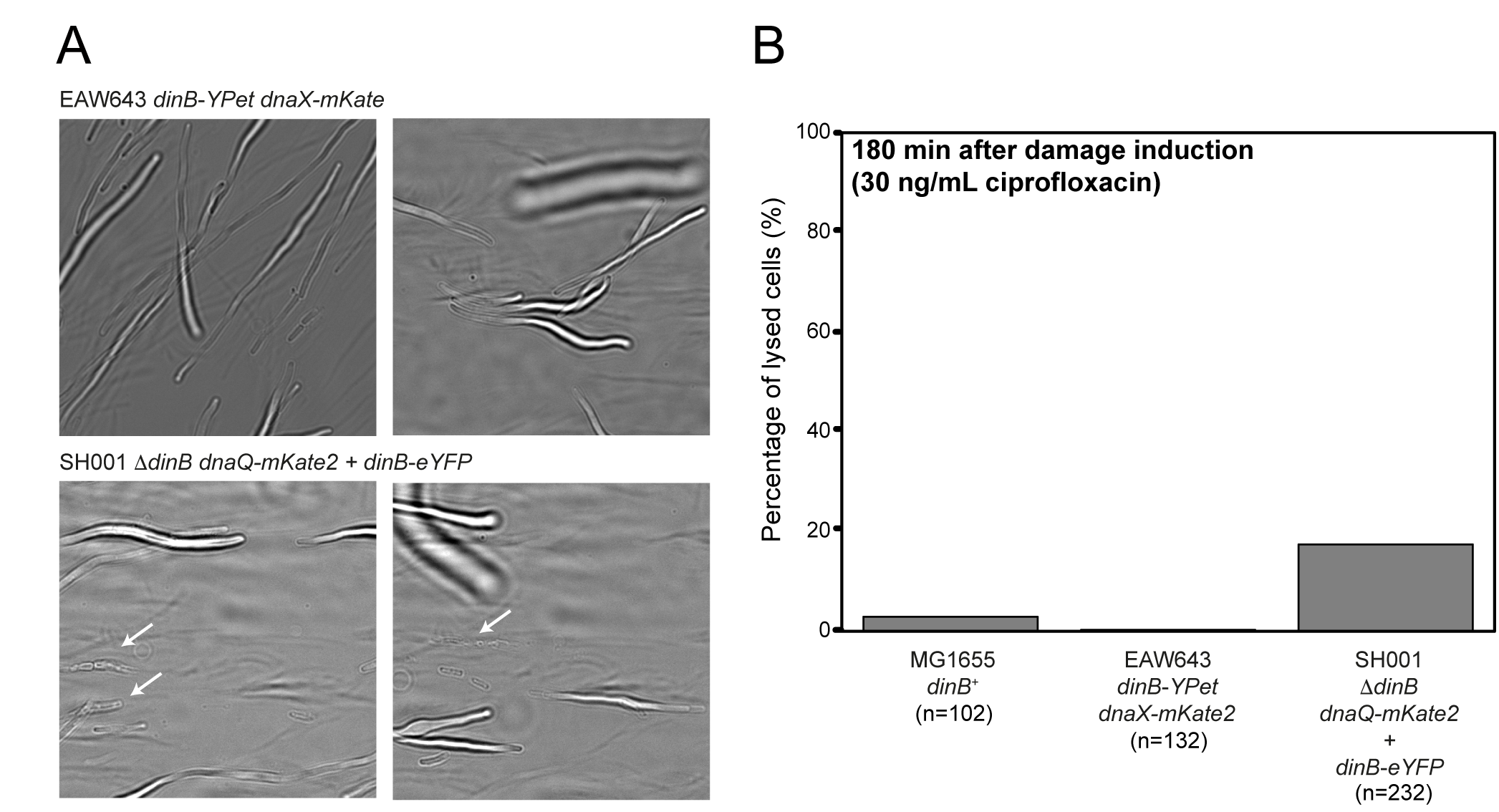

Supplement: S7 Fig — (A) Representative bright-field images of EAW643 cells (top two panels) and SSH001 pPFB1188 cells (bottom two panels), 180 min after the addition of 30 ng/ml ciprofloxacin. Arrows indicate the positions of cells that have lysed. (B) Bar graph showing the percentage of cells that lyse by the 180 min time-point for MG1655, EAW643 and SSH001 pPFB1188 cells. The number of cells that were tracked were as follows: MG1655, 102 cells; EAW643, 132 cells; SSH001 pPFB1188, 232 cells. (TIF) [file pgen.1007161.s007.tif]

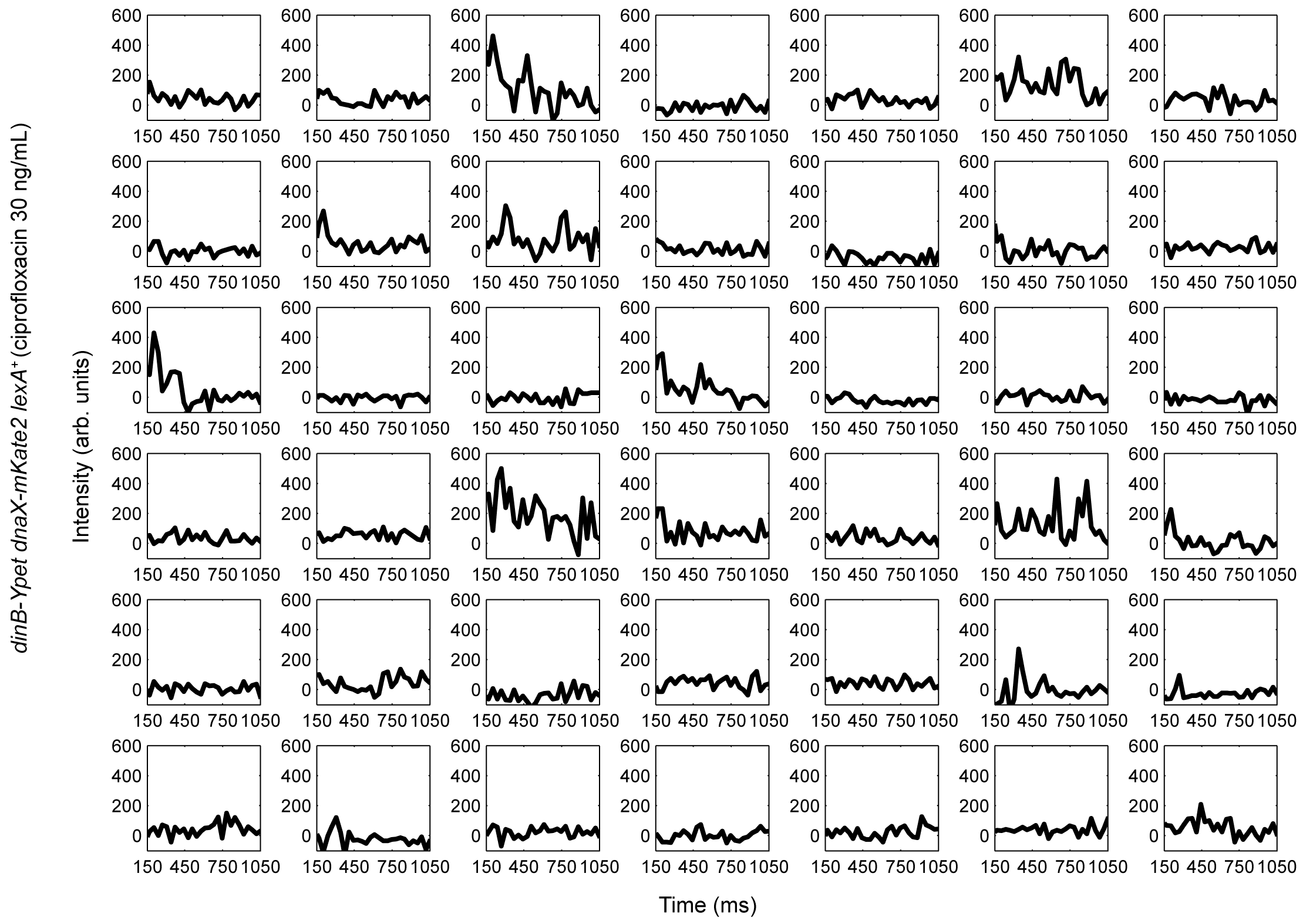

Supplement: S8 Fig — 5×5 pixel regions of interest were placed at replisome foci, then used to monitor fluctuations in DinB-YPet signals (see Fig 6A). A subset of 42 trajectories were selected randomly from a total of 470 trajectories. To allow comparison with DinB-YPet singles in lexA(Def) cells, where expression levels are too high to observe single-molecule foci, we present only a portion of each trajectory, starting at a time-point (150 ms) where ~50% of DinB-YPet molecules have already photobleached. In ciprofloxacin-treated EAW643 cells, DinB-YPet signals are frequently elevated in the vicinity of replisomes for multiple frames, indicating long-lived binding events. (TIF) [file pgen.1007161.s008.tif]

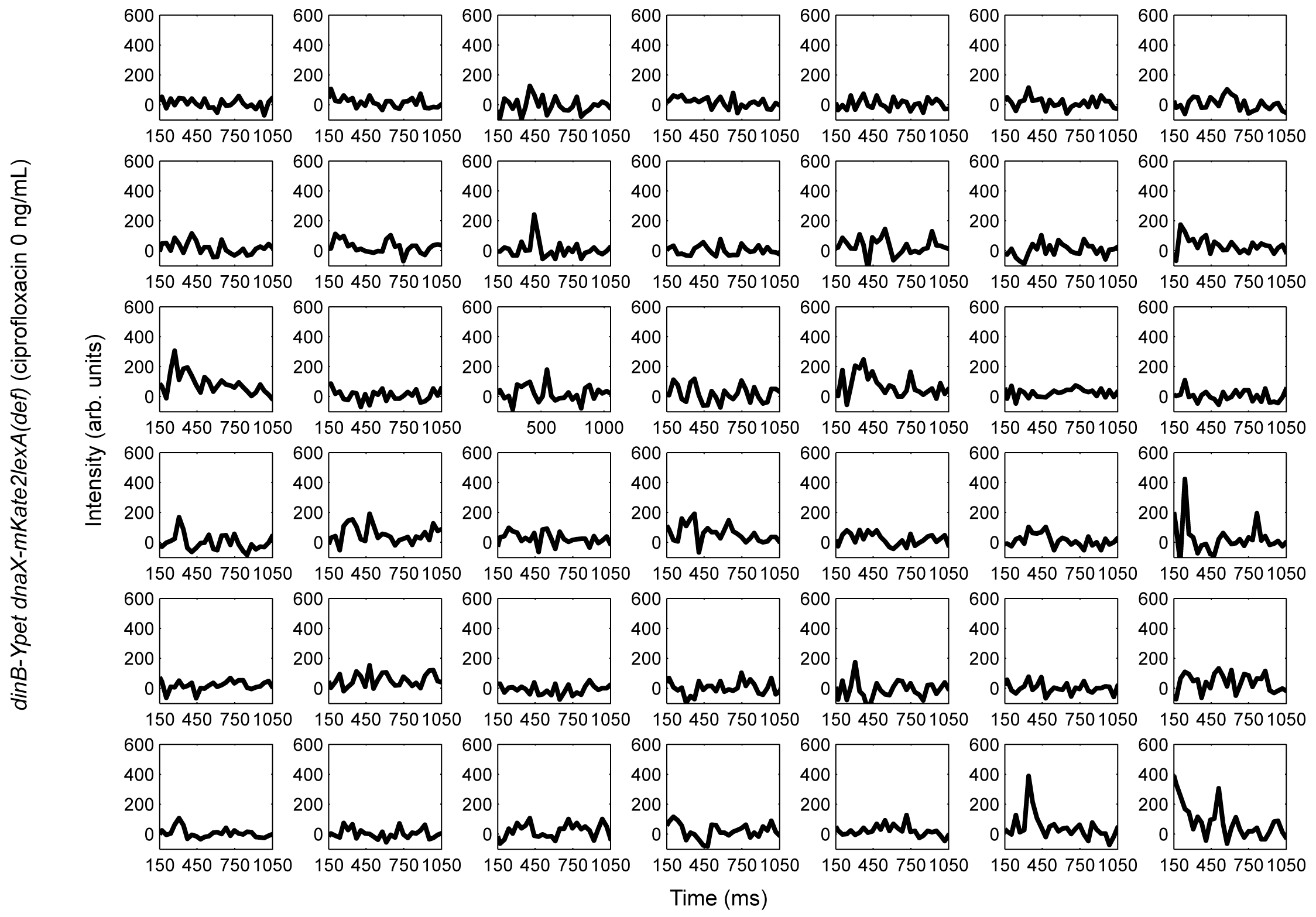

Supplement: S9 Fig — 5×5 pixel regions of interest were placed at replisome foci, then used to monitor fluctuations in DinB-YPet signals (see Fig 6A). A subset of 42 trajectories were selected randomly from a total of 65 trajectories. A portion of each trajectory is presented, starting at a time-point (150 ms) where ~50% of DinB-YPet molecules have already photobleached, allowing single-molecule foci to be observed. In untreated lexA(Def) cells few events are visible in which the DinB-YPet is elevated in the vicinity of replisomes for more than a single 34 ms frame, indicating few long-lived binding events. (TIF) [file pgen.1007161.s009.tif]

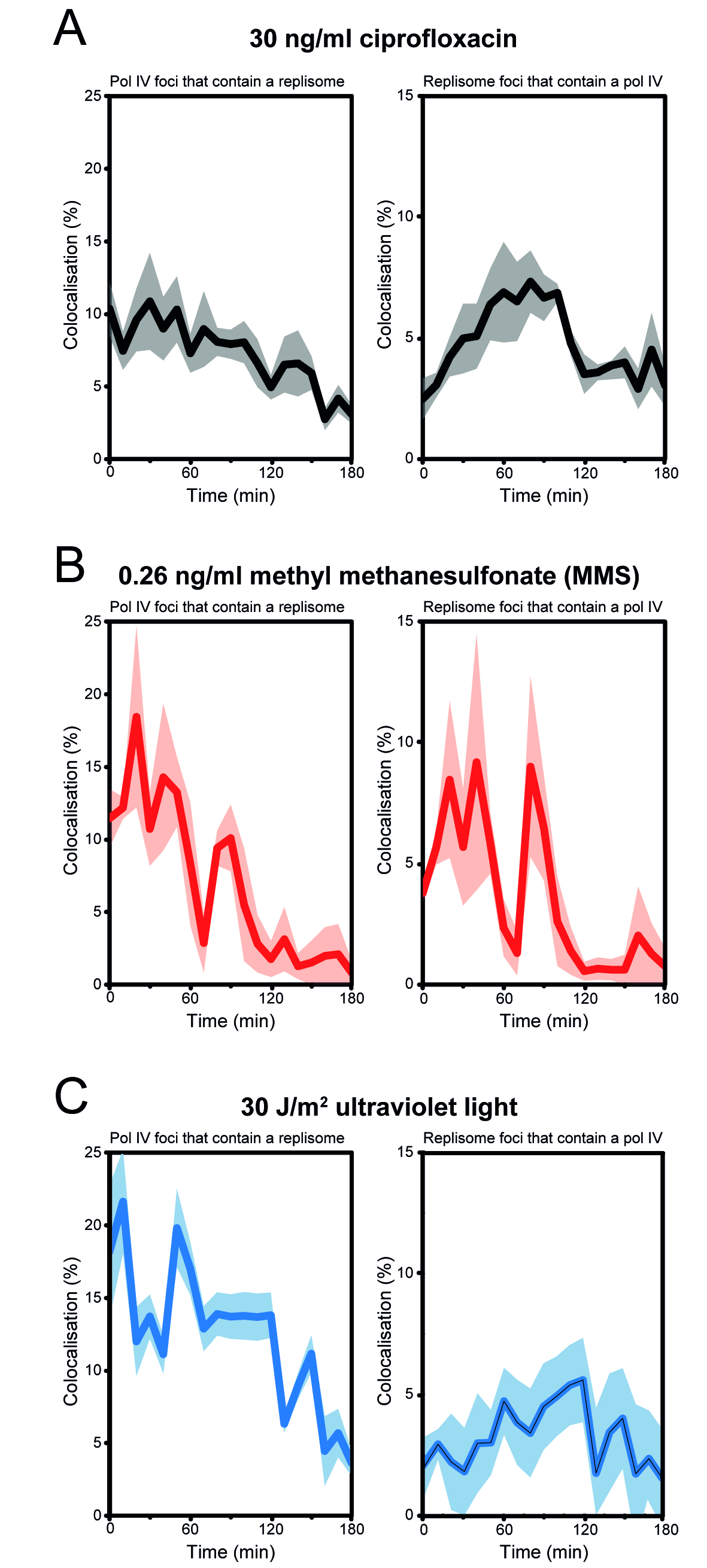

Supplement: S10 Fig — Comparison of pol IV-replisome colocalisation in cells treated with different DNA-damaging agents: (A) 30 ng/ml ciprofloxacin, (B) 0.26 ng/ml methyl methanesulfonate, (C) ultraviolet light (fluence = 30 J/m2, flux density = 3.3 W/m2, λ = 254 nm). Colocalisation (A–C) was measured in two ways: the proportion of DinB-YPet foci that contain a colocalised DnaX-mKate2 (replisome) focus, and the proportion of DnaX-mKate2 foci that contain a colocalised DinB-YPet focus. Shaded areas indicate the standard error of the proportion. All DNA-damaging agents produce a distinctive drop in colocalisation 90–120 min after treatment. We conservatively estimate that >200 cells were used in each measurement. (TIF) [file pgen.1007161.s010.tif]

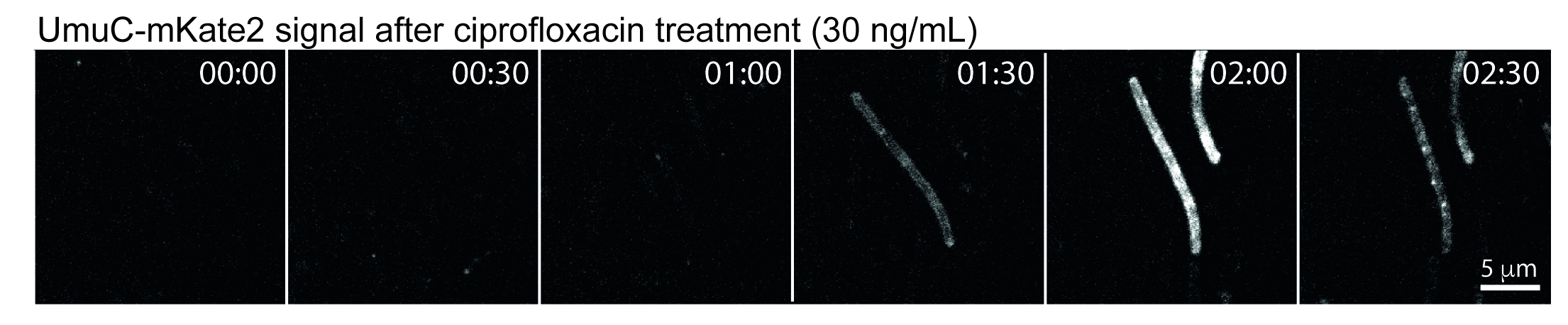

Supplement: S11 Fig — We previously discovered that pol V is spatially regulated: the UmuC protein accumulates at the cell membrane, until the active form pol V Mut (UmuD′2-UmuC-RecA-ATP) is formed and released into the cytosol [47]. This was monitored by observing the change in cellular localisation of UmuC-mKate2 as a function of time. When cells are instead treated with ciprofloxacin, UmuC-mKate2 goes through a very similar progression of localisation states. In the example shown in this figure, UmuC-mKate2 is initially absent (0–1 h), then membrane associated (1.5–2 h), then cytosolic (2.5–3 h). The timing of these transitions varies from cell to cell. Typically, no UmuC-mKate2 is visible until 45–90 min after ciprofloxacin treatment. From 45–150 min, UmuC-mKate2 is membrane-associated. UmuC-mKate2 typically remains membrane-associated for approximately 30 min before being released into the cytosol. (TIF) [file pgen.1007161.s011.tif]
